# Genetic dissection of triplicated chromosome 21 orthologs yields varying skeletal traits in Down syndrome model mice

**DOI:** 10.1242/dmm.049927

**Published:** 2023-04-26

**Authors:** Kourtney Sloan, Jared Thomas, Matthew Blackwell, Deanna Voisard, Eva Lana-Elola, Sheona Watson-Scales, Daniel L. Roper, Joseph M. Wallace, Elizabeth M. C. Fisher, Victor L. J. Tybulewicz, Randall J. Roper

**Affiliations:** ^1^ Department of Biology, Indiana University-Purdue University Indianapolis, Indianapolis, IN 46202, USA; ^2^ The Francis Crick Institute, London NW1 1AT, UK; ^3^ Data Analytics Computing, Lehi, UT 84043, USA; ^4^ Department of Biomedical Engineering, Indiana University-Purdue University Indianapolis, Indianapolis, IN 46202, USA; ^5^ UCL Institute of Neurology, London WC1N 3BG, UK

**Keywords:** Down syndrome, Trisomy 21, Skeletal deficits, Animal models, Genetics

## Abstract

Down syndrome (DS) phenotypes result from triplicated genes, but the effects of three copy genes are not well known. A mouse mapping panel genetically dissecting human chromosome 21 (Hsa21) syntenic regions was used to investigate the contributions and interactions of triplicated Hsa21 orthologous genes on mouse chromosome 16 (Mmu16) on skeletal phenotypes. Skeletal structure and mechanical properties were assessed in femurs of male and female Dp9Tyb, Dp2Tyb, Dp3Tyb, Dp4Tyb, Dp5Tyb, Dp6Tyb, Ts1Rhr and Dp1Tyb;*Dyrk1a*^+/+/−^ mice. Dp1Tyb mice, with the entire Hsa21 homologous region of Mmu16 triplicated, display bone deficits similar to those of humans with DS and served as a baseline for other strains in the panel. Bone phenotypes varied based on triplicated gene content, sex and bone compartment. Three copies of *Dyrk1a* played a sex-specific, essential role in trabecular deficits and may interact with other genes to influence cortical deficits related to DS. Triplicated genes in Dp9Tyb and Dp2Tyb mice improved some skeletal parameters. As triplicated genes can both improve and worsen bone deficits, it is important to understand the interaction between and molecular mechanisms of skeletal alterations affected by these genes.

## Teaser

**Summary:** A Down syndrome mouse mapping panel shows that triplicated *Dyrk1a* plays a sex-specific role in some skeletal phenotypes. Triplicated genes may interact and cause improved or worsened skeletal measurements.

## INTRODUCTION

The genotype–phenotype etiology of Down syndrome (DS), or trisomy 21 (Ts21), affecting ∼1 in 800 live births (de Graaf et al., 2017), is not well understood. Trisomy of human chromosome 21 (Hsa21) produces a dosage imbalance of the genes on Hsa21, which can result in altered protein levels to cause deficits in cognitive, skeletal, cardiac, immune and other systems seen in DS. Multiple non-mutually exclusive hypotheses describe the potential effect of three copy Hsa21 genes on DS phenotypes: a single ‘effector’ or ‘driver’ triplicated gene may play a major role in a particular phenotype and may affect other triplicated or non-triplicated responder genes that cause particular DS phenotypes; multiple triplicated genes may interact to cause a DS phenotype; or compensatory effects may mask the effect of three copy Hsa21 genes and their role in a phenotype (Roper and Reeves, 2006; Antonarakis et al., 2020; Moyer et al., 2021; Duchon et al., 2021). These hypothesized genetic mechanisms may be different for each DS phenotype. The possibility that Ts21 disrupts normal gene expression also leads to questions about the mechanistic manifestation of a particular phenotype or how three copies of ‘dosage-sensitive’ genes influence subphenotypes of a DS trait, including whether triplicated genes have both positive and negative effects on a phenotype or subphenotype (Moyer et al., 2021). Insights into these questions will help understand the effects of genetic dosage imbalance and potential correction of temporally and spatially altered gene expression to improve DS traits.

Significant differences in bone mineral density (BMD) have been observed in individuals with DS beginning in their second and third decades of life. Individuals with DS attain peak bone mass 5-10 years earlier than the general population, and their peak bone mass is lower than that of the general population (Costa et al., 2018). Additionally, people with DS experience bone loss sooner and at a higher rate than the general population (Carfì et al., 2017). Males with DS begin losing BMD in the femur much earlier than females with DS, suggesting a protective effect of the Hsa21 trisomy in the female biological sex in terms of maintaining BMD (Carfì et al., 2017; Costa et al., 2017, 2018; Tang et al., 2019). Lowered BMD is thought to be the product of dysregulated bone turnover and may be due to low bone formation, which has been shown in people with DS through serum biomarkers (McKelvey et al., 2013).

Mouse models are commonly used to study DS phenotypes, and the availability of strains with different segments of Hsa21 orthologous genes in three copies allows mapping of the specific dosage-sensitive genes that cause particular phenotypic outcomes (Antonarakis et al., 2020; Lana-Elola et al., 2011, 2016; Duchon et al., 2021). Genes orthologous to Hsa21 are located on mouse chromosome (Mmu)16, Mmu17 and Mmu10, with the greatest number being located distally on Mmu16 (Davisson et al., 2001). DS skeletal phenotypes, reflecting changes in both males and females, have been recapitulated in DS mouse models including Ts65Dn (∼50% of Hsa21 orthologous genes on a freely segregating extra chromosome) and Dp1Tyb (duplication of 145 protein-coding genes and 23 Mb on Mmu16 that is orthologous to Hsa21) (Blazek et al., 2011; Lana-Elola et al., 2021, 2016; Thomas et al., 2020; Thomas and Roper, 2021).

Dual-specificity tyrosine phosphorylation-regulated kinase 1A (*DYRK1A*), found in three copies in individuals with DS and some DS mouse models, including Ts65Dn and Dp1Tyb, is hypothesized to affect multiple areas of development, including cognitive and skeletal systems. Normalizing *Dyrk1a* copy number in Ts65Dn;*Dyrk1a*^+/+/−^ male mice at 6 weeks of age improved trabecular and cortical microarchitecture, mechanical and cellular properties, indicating that *Dyrk1a* copy number is important in skeletal properties of male mice (Blazek et al., 2015). However, other triplicated genes in addition to *Dyrk1a* may also be important in skeletal phenotypes and specifically in female DS mouse models.

Ts1Rhr mice have three copies of 31 protein-coding genes (including *Dyrk1a*) and 4.2 Mb of Mmu16 that is orthologous to Hsa21 (Olson et al., 2004, 2007). Limited skeletal measurements found only small changes in Ts1Rhr mouse bone compared to that of control littermates, and no skeletal differences between male and female Ts1Rhr mice; trabecular, cortical and mechanical parameters were never quantified in these mice (Olson and Mohan, 2011). Tg(*DYRK1A*) mice with extra *DYRK1A* copies showed trabecular deficits in both male and female mice but no deficits in cortical thickness (Ct.Th) (other cortical properties were not examined) (Lee et al., 2009). These results led to questions pertaining to the importance of increased *Dyrk1a* copy number and sexual dimorphism in DS-related trabecular and cortical bone deficits.

To facilitate the identification of important dosage-sensitive genes that lead to DS phenotypes, including DS-related cardiac and locomotor deficits, a high-resolution mapping panel of seven strains, with contiguous genetic segmental duplications covering various regions of Mmu16 that correspond to Hsa21, was generated (Lana-Elola et al., 2016; Watson-Scales et al., 2018). We utilized this mouse mapping panel to identify triplicated genes or regions that were important in bone phenotypes. We hypothesized that mouse lines containing triplicated *Dyrk1a* (Dp3Tyb and Dp5Tyb) would have more severe deficits in skeletal phenotypes than those of other triplicated lines, and that sexual dimorphisms would be seen in bone deficits. Coupled with results from Ts1Rhr and Dp1Tyb;*Dyrk1a*^+/+/−^ mice, we more accurately defined the contribution of three copies of *Dyrk1a* to deficits in various bone compartments in a sex-specific manner and identified potential interacting genes that lead to skeletal phenotypes associated with DS.

## RESULTS

### Different segments of Hsa21 orthologous genes alter trabecular microarchitecture in diverse ways, with three copies of *Dyrk1a* influencing trabecular bone in a sex-dependent manner

Male Dp1Tyb compared to control littermate mice displayed reduced BMD, bone volume fraction (BV/TV) and trabecular thickness (Tb.Th) at 16 weeks (4 months) (Thomas et al., 2020). Female 16-week-old Dp1Tyb and control mice had similar skeletal measurements for BMD, BV/TV, trabecular number (Tb.N), Tb.Th and trabecular separation (Tb.Sp). There were sex effects on skeletal deficits, as male 16-week-old Dp1Tyb and control mice displayed higher BMD, BV/TV and Tb.N, and lower Tb.Sp, than those of female Dp1Tyb and control mice at the same age.

**Fig. 1.**
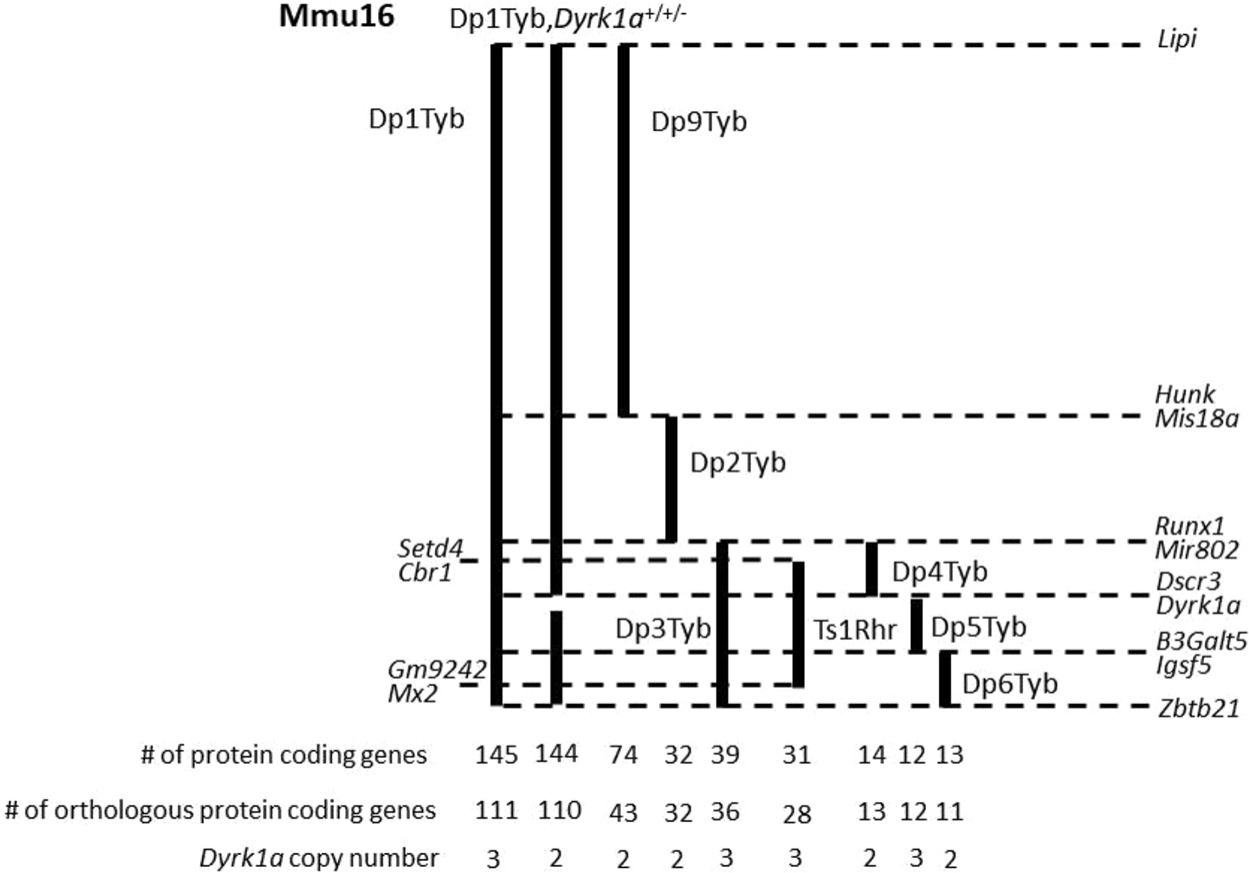
**Mouse mapping panel of triplicated regions of the Hsa21 orthologous genes on mouse chromosome 16 (Mmu16).** The solid lines indicate the extent of the triplicated regions, with the first and last genes included in each triplication on the right; dashed lines denote boundaries between triplicated regions in different strains. The numbers of coding genes are based on the GRCm39 mouse genome assembly and have changed slightly from the numbers reported in Lana-Elola et al. (2016) due to changes in genome annotation.

**Fig. 2.**
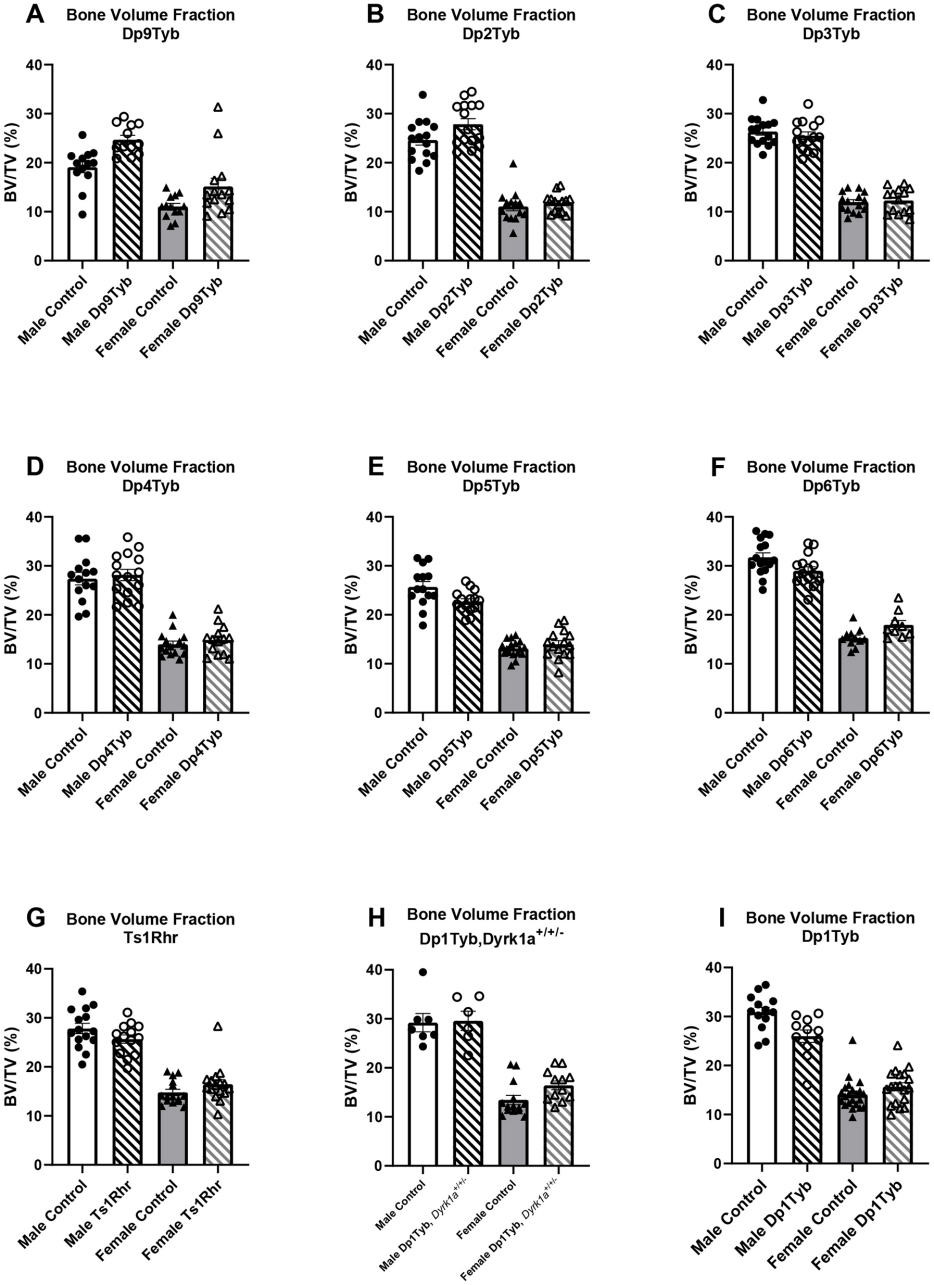
**Bone volume fraction (BV/TV) measurements in triplicated (Dp) mouse models and control mice.** Animal numbers are as listed in Tables 1 and 2. Data are mean±s.e.m. Dp1Tyb data are from Thomas et al. (2020).

To better comprehend how three copies of Hsa21 orthologous genes on Mmu16 affect trabecular bone, 4-month-old Dp9Tyb, Dp2Tyb and Dp3Tyb mouse lines that divide the Dp1Tyb region of duplication of Mmu16 into three, non-overlapping segments were analyzed (Fig. 1). Male and female, triplicated and control mice were examined together within each line. There was a significant effect of sex, with more ‘positive’ effects on trabecular parameters in male than in female mice (together) for the Dp9Tyb, Dp2Tyb and Dp3Tyb mouse lines (Fig. 2A-C, Table 1; Fig. S1A-C, Fig. S2A-C, Fig. S3A-C, Fig. S4A-C). Unexpectedly, there were significant genotype effects in Dp9Tyb and Dp2Tyb lines compared to controls, with increased BMD, BV/TV, Tb.N and Tb.Th, and decreased Tb.Sp, in the Dp9Tyb compared to the control mice, and increased Tb.N and decreased Tb.Sp in the Dp2Tyb compared to the control mice. Dp3Tyb mice did not show any significant genotype effects for trabecular parameters.

**Table 1.**
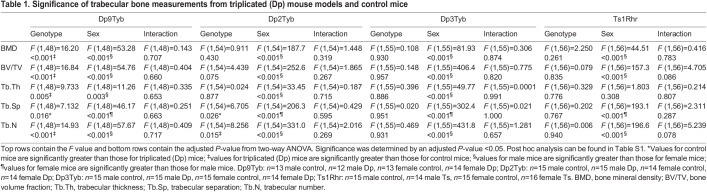
Significance of trabecular bone measurements from triplicated (Dp) mouse models and control mice

**Table 2.**
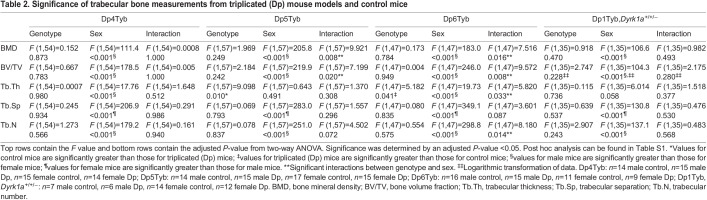
Significance of trabecular bone measurements from triplicated (Dp) mouse models and control mice

Given previous work showing the influence of trisomic *Dyrk1a* on bone, it was surprising to find no genotypic effect in the Dp3Tyb line. To better understand the contribution of triplicated genes from the Dp3Tyb region on trabecular phenotypes associated with DS, we analyzed the Dp4Tyb, Dp5Tyb and Dp6Tyb lines (including littermate controls) that split the Dp3Tyb duplication into three separate regions, with only Dp5Tyb mice containing three copies of *Dyrk1a* (Fig. 1). At 4 months, sex effects were found in trabecular parameters for the Dp4Tyb line, where male mice had increased BMD, BV/TV, Tb.Th and Tb.N, and decreased Tb.Sp, compared to those of female mice (Fig. 2D, Table 2; Fig. S1D, Fig. S2D, Fig. S3D, Fig. S4D). There were interactive effects for trabecular traits between sex and genotype for the Dp5Tyb (BMD and BV/TV) and Dp6Tyb (BMD, BV/TV, Tb.Th and Tb.N) lines compared to their respective littermate controls (Fig. 2E,F, Table 2; Fig. S1E,F, Fig. S2E,F, Fig. S3E,F, Fig. S4E,F). Male Dp5Tyb and Dp6Tyb and littermate control mice displayed greater BMD and BV/TV measurements than those of female Dp5Tyb and Dp6Tyb and littermate control mice, respectively. Only male Dp5Tyb mice, compared to male control mice, showed decreased BMD in post hoc tests, suggesting that three copies of *Dyrk1a* (or another gene in the Dp5Tyb region) are necessary to significantly alter BMD in male, but not female, mice (Table S1).

Furthermore, an interactive effect was observed for Tb.Th, where female Dp6Tyb mice, compared to control mice, showed an increase in Tb.Th in post hoc tests (Table 2; Table S1). There was also a genotype effect, with Dp5Tyb displaying decreased Tb.Th compared to that in control mice (Table 2). Taken together, these data suggest complex and sex-specific influences of triplicated genes (possibly including *Dyrk1a*) affecting Tb.Th in the genetic regions covered by Dp5Tyb and Dp6Tyb lines.

Additionally, Ts1Rhr mice (three copies of 31 protein-coding genes, including *Dyrk1a*, with eight fewer protein-coding genes than Dp3Tyb mice) displayed sex differences in trabecular parameters (Fig. 2G, Table 1; Fig. S1G, Fig. S2G, Fig. S3G, Fig. S4G). In the Ts1Rhr line, male mice had higher BMD and Tb.N, and lower Tb.Sp, than those of female mice. Finally, the Dp1Tyb;*Dyrk1a*^+/+/−^ line was generated to observe the effects of normalizing *Dyrk1a* copy number from conception in an otherwise Dp1Tyb mouse. There were no genotypic effects in any trabecular parameter in Dp1Tyb;*Dyrk1a*^+/+/−^ mice compared to control (wild-type) animals (Fig. 2H, Table 2; Fig. S1H, Fig. S2H, Fig. S3H, Fig. S4H); this normalization of phenotypes in Dp1Tyb mice with only two functional copes of *Dyrk1a* shows the importance of triplicated *Dyrk1a* in trabecular phenotypes. Sex effects were observed in the Dp1Tyb;*Dyrk1a*^+/+/−^ line, with male mice displaying higher BV/TV, BMD and Tb.N, and lower Tb.Sp, than those of female mice. Taken together, these data suggest that three copies of *Dyrk1a* are necessary, but not sufficient, to decrease BV/TV, BMD and Tb.Th, and interact with other triplicated genes from the Dp5Tyb and Dp6Tyb regions to cause trabecular deficits in a sex-specific manner.

### Cortical geometry parameters differ according to differential segments of Hsa21 orthologous genes in three copies

At 16 weeks of age (4 months), total (cortical) cross-sectional area (Tt.Ar) was larger in control male mice than in all other mice, in male Dp1Tyb mice than in female Dp1Tyb mice, and in female control mice than in female Dp1Tyb mice (Thomas et al., 2020). At 16 weeks of age, male control mice had larger Tt.Ar, cortical area (Ct.Ar), periosteal perimeter (Ps.Pm) and endocortical perimeter (Ec.Pm) than those of all other mice, with male mice as a group having higher parameters than those of female mice. Marrow area (Ma.Ar) was greater in male control mice than in all other mice, and in female control mice than in female Dp1Tyb mice. Female mice as a group had lower Ct.Th than that of male mice.

**Fig. 3.**
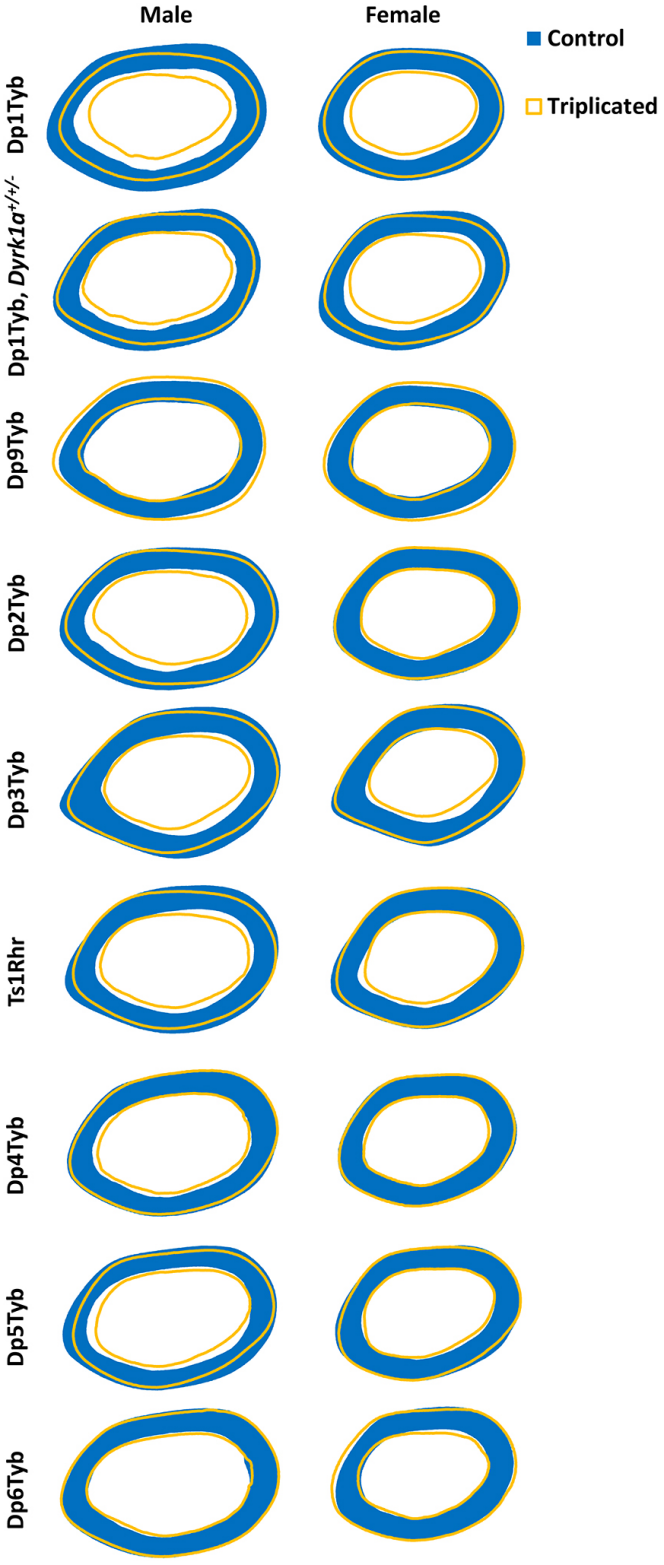
**Cortical models of triplicated (Dp) mouse models and control mice.** Animal numbers are as listed in Tables 1 and 2. Dp1Tyb data are from Thomas et al. (2020). These radar graphs were made using average periosteal and endocortical perimeter measurements taken every 0.5° for each cortical slice (total of seven) of each animal. The blue, filled-in images represent the average cross-section of male or female control animals, and the yellow outlines represent the average cross-section of male or female triplicated animals.

**Fig. 4.**
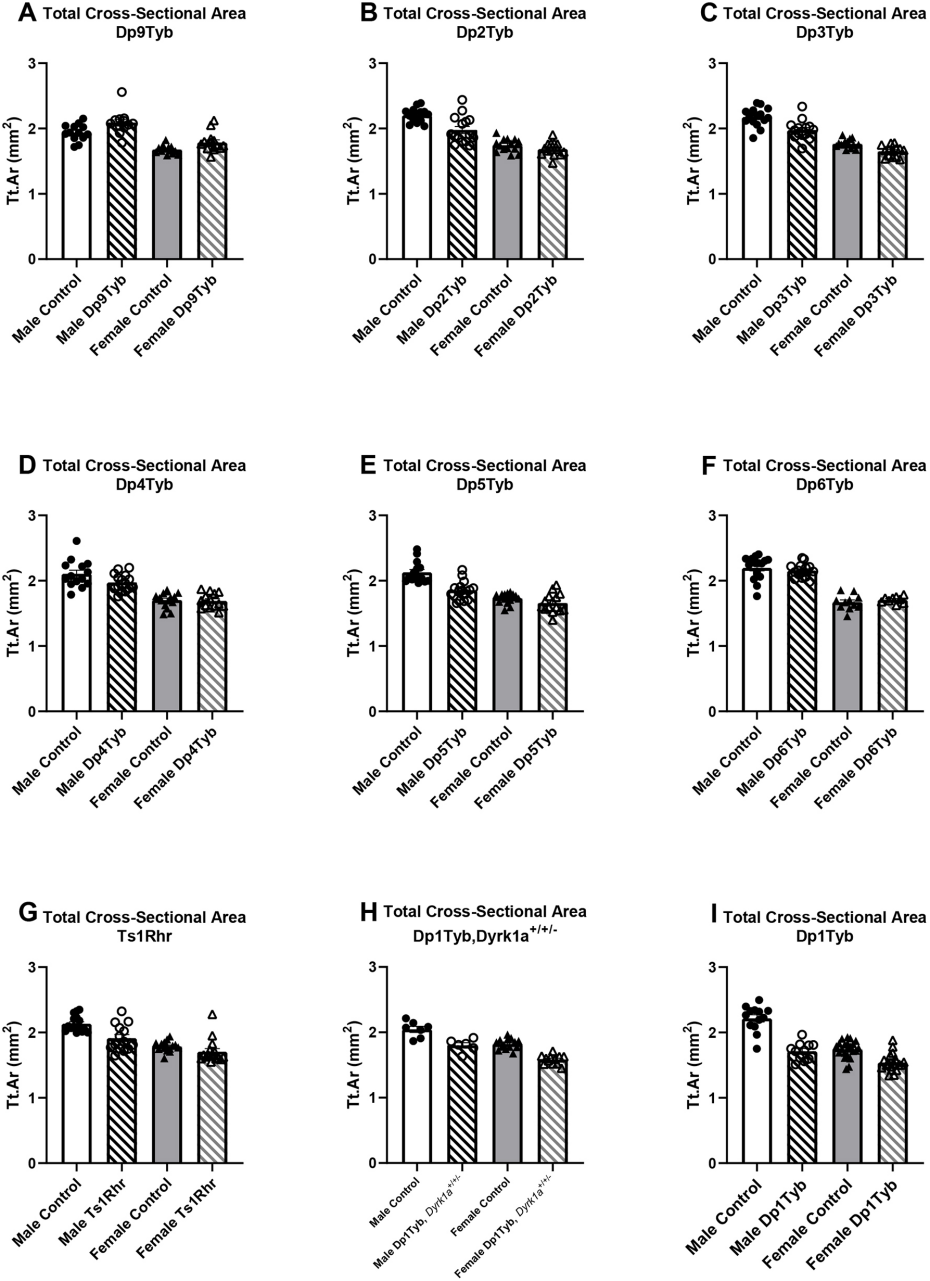
**Total cross-sectional area (Tt.Ar) measurements in triplicated (Dp) mouse models and control mice.** Animal numbers are as listed in Tables 1 and 2. Data are mean±s.e.m. Dp1Tyb data are from Thomas et al. (2020).

At 4 months of age, sex effects were observed in the Dp9Tyb line, with male mice displaying higher Tt.Ar, Ma.Ar, Ct.Ar, Ps.Pm and Ec.Pm, and lower cortical tissue mineral density (TMD), than those of female mice. There were genotype effects, with Dp9Tyb mice with their triplicated genes unexpectedly showing increased Tt.Ar, Ma.Ar, Ct.Ar, Ps.Pm and Ec.Pm compared to those of control mice (Fig. 3, Fig. 4A, Table 3; Fig. S5A, Fig. S6A, Fig. S7A, Fig. S8A, Fig. S9A, Fig. S10A). Dp2Tyb and control mice showed significant interactions between sex and genotype, with male Dp2Tyb and control mice displaying higher Tt.Ar, Ma.Ar and Ec.Pm than those of female Dp2Tyb and control mice (Fig. 3, Fig. 4B, Table 3; Fig. S5B, Fig. S6B, Fig. S7B, Fig. S8B, Fig. S9B). Male control mice, compared to male Dp2Tyb mice, showed increased Tt.Ar, Ma.Ar and Ec.Pm in post hoc analysis, suggesting that triplicated gene(s) in this region act in a sex-specific manner to decrease these cortical parameters (Table S1). Additionally, sex effects were present in the Dp2Tyb line, where male mice had increased Ct.Ar, Ct.Th and Ps.Pm, and decreased TMD, compared to those of female mice (Fig. 3, Table 3; Fig. S6B, Fig. S7B, Fig. S8B, Fig. S10B). Differing genotypic effects were seen for Ct.Th and Ps.Pm, where Dp2Tyb mice had increased Ct.Th, but decreased Ps.Pm, compared to that of control mice (Fig. 3, Table 3; Fig. S7B, Fig. S8B). In animals from the Dp3Tyb line, there were sex effects, with male mice displaying greater Tt.Ar, Ma.Ar, Ct.Ar, Ct.Th, Ps.Pm and Ec.Pm than those of female mice. Additionally, there were genotype effects, with Dp3Tyb mice showing significantly lower Tt.Ar, Ma.Ar, Ct.Ar, Ps.Pm and Ec.Pm than those of control mice (Fig. 3, Fig. 4C, Table 3; Fig. S5C, Fig. S6C, Fig. S7C, Fig. S8C, Fig. SS9C). There was also an interactive effect of sex and genotype on TMD, with male control and Dp3Tyb mice displaying increased TMD compared to that of female Dp3Tyb mice (Table 3; Fig. S10, Table S1). These results suggest that different triplicated regions may increase (Dp9Tyb) or decrease (Dp2Tyb and Dp3Tyb) cortical phenotypes.

**Table 3.**
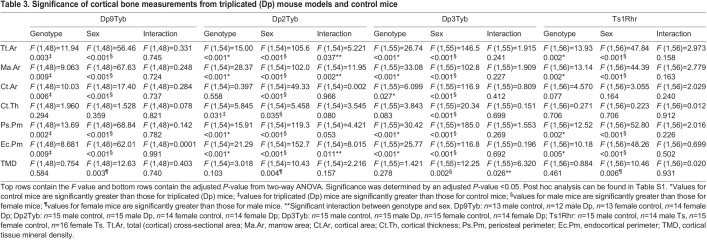
Significance of cortical bone measurements from triplicated (Dp) mouse models and control mice

To understand the impact of the triplicated genes comprising the Dp3Tyb region on cortical phenotypes, Dp4Tyb, Dp5Tyb, Dp6Tyb and control littermate mice were analyzed. Sex effects were observed in the Dp4Tyb and Dp6Tyb lines, with male mice exhibiting greater Tt.Ar, Ma.Ar, Ct.Ar, Ps.Pm and Ec.Pm, and lower TMD, than those of female mice (Fig. 3, Fig. 4D,F, Table 4; Fig. S5D,F, Fig. S6D,F, Fig. S7D,F, Fig. S8D,F, Fig. S9D,F, Fig. S10D,F). Additionally, there was a genotypic effect for Ma.Ar in the Dp4Tyb line, where control mice exhibited greater Ma.Ar than that of Dp4Tyb mice. The Dp5Tyb line (containing three copies of *Dyrk1a*) showed significant interactions in all cortical parameters measured except Ct.Th and TMD, for which only sex effects and genotype effects were observed. Similar to trabecular bone, only male Dp5Tyb mice, compared to male control mice, showed decreased Tt.Ar, Ma.Ar, Ct.Ar, Ps.Pm and Ec.Pm in post hoc tests (Fig. 3, Fig. 4E, Table 4; Fig. S5E, Fig. S6E, Fig. S8E, Fig. S9E, Table S1). For most parameters, both male Dp5Tyb and control mice had significantly greater measurements than those of female Dp5Tyb and control mice in post hoc tests, suggesting that three copies of *Dyrk1a* or other triplicated genes in this region are necessary to significantly alter Tt.Ar, Ma.Ar, Ct.Ar, Ps.Pm and Ec.Pm in male, but not female, mice (Table S1).

**Table 4.**
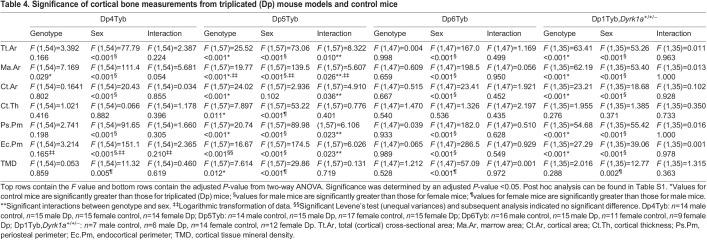
Significance of cortical bone measurements from triplicated (Dp) mouse models and control mice

The Ts1Rhr line displayed similar cortical parameters to the Dp3Tyb line, with the exception of no significant differences in Ct.Ar or Ct.Th, and an opposite sex effect for TMD (Fig. 3, Fig. 4G, Table 3; Fig. S5G, Fig. S6G, Fig. S7G, Fig. S8G, Fig. S9G, Fig. S10G). Normalizing *Dyrk1a* copy number in Dp1Tyb;*Dyrk1a*^+/+/−^ mice did not normalize cortical parameters and still resulted in significant differences in Tt.Ar, Ma.Ar, Ct.Ar, Ps.Pm and Ec.Pm according to sex and genotype (Fig. 3, Fig. 4H, Table 4; Fig. S5H, Fig. S6H, Fig. S7H, Fig. S8H, Fig. S9H). Also, TMD was higher in female mice than in male mice in the Dp1Tyb;*Dyrk1a*^+/+/−^ line (Table 4; Fig. S10H). Male mice with three copies of *Dyrk1a* (Dp3Tyb, Dp5Tyb and Ts1Rhr) showed significant percentage decreases in Tt.Ar, Ma.Ar, Ps.Pm and Ec.Pm, compared to those of control mice, but these percentage decreases were generally not of the same magnitude as those in male Dp1Tyb mice (Table S3). Female mice with the Dp3Tyb region in three copies had significant percentage decreases in Tt.Ar, Ma.Ar, Ps.Pm and Ec.Pm compared to those of control mice (Tables S3). Male mice with three copies of the Dp2Tyb region also showed a reduction in the percentage decreases in multiple cortical parameters compared to those of control mice (Table S3). Taken together, these data indicate that the effects of three copies of *Dyrk1a* are important in reducing many cortical parameters, but there is an interactive effect of sex, with cortical parameters mostly affected in male mice. Other triplicated genes inside and outside the Dp3Tyb region may interact with *Dyrk1a* to cause differences in cortical parameters between triplicated and normal mice, and there may be different interacting genes involved in male and female mice with three copies of Hsa21 homologs.

### Whole-bone property differences lessen in the presence of a smaller number of Hsa21 orthologous genes in three copies

Alterations in extrinsic mechanical properties, based on bone mass and cortical geometry, were observed in 16-week-old (4-month-old) Dp1Tyb mice (Thomas et al., 2020). At 16 weeks of age, there was a sex×genotype interaction for ultimate force, with control males having greater values than those of all other mice and Dp1Tyb males having greater values than those of Dp1Tyb females. Control, compared to Dp1Tyb, mice had higher values for ultimate displacement, stiffness and total work; Dp1Tyb, compared to control, mice displayed higher values for displacement to yield and work to yield. Also, males, compared to females, had higher measurements for ultimate displacement, stiffness and total work. At 16 weeks, sex, and especially genotype, had non-interactive effects on overall bone properties, and additional gene dosage and female sex were detrimental to whole-bone properties.

Comparisons were made between the Dp9Tyb, Dp2Tyb and Dp3Tyb lines that divide the triplicated region of Dp1Tyb. In the Dp9Tyb line, there was a genotypic effect on ultimate force, where Dp9Tyb mice could handle a greater force than control mice could (Table 5). Sex effects were also observed in the Dp9Tyb line, where female mice had higher measurements for yield force, displacement to yield and work to yield than those of male mice, suggesting they have a greater elastic region. In the Dp2Tyb line, there was a genotypic effect on ultimate displacement, with control mice having greater displacement than Dp2Tyb mice (Table 5). Additionally, there were sex effects, with male mice showing greater values for ultimate force and ultimate displacement, but lower values for yield force, displacement to yield and work to yield, than female mice. The Dp3Tyb line showed sex effects only, where male mice had higher values for ultimate force and ultimate displacement, but lower values for displacement to yield and work to yield, than those of female mice (Table 5). A sex effect on ultimate displacement was also observed in Dp3Tyb mice, with male mice having higher values than those of female mice.

**Table 5.**
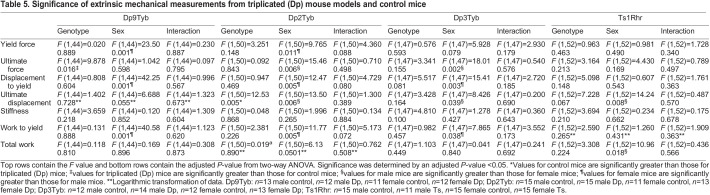
Significance of extrinsic mechanical measurements from triplicated (Dp) mouse models and control mice

To understand the potential impact of triplicated genes in the Dp3Tyb region on extrinsic properties in bone, we examined the Dp4Tyb, Dp5Tyb and Dp6Tyb lines, in which the Dp3Tyb region is split (Table 6). The Dp4Tyb and Dp5Tyb lines showed no significant differences in any extrinsic parameter. The Dp6Tyb line showed only sex effects, with female mice displaying higher values for displacement to yield and work to yield than those of male mice, and male mice displaying higher values for ultimate displacement, stiffness and total work than those of female mice.

**Table 6.**
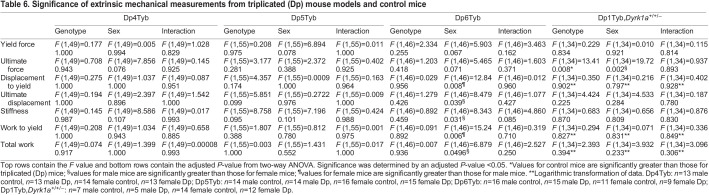
Significance of extrinsic mechanical measurements from triplicated (Dp) mouse models and control mice

Ts1Rhr mice showed sex effects on ultimate displacement and total work, with male mice having higher values than those of female mice (Table 5). Extrinsic differences previously seen in Dp1Tyb mice, except for sex and genotype effects on ultimate force, were not observed in Dp1Tyb;*Dyrk1a*^+/+/−^ mice (Table 6).

### Material property improvements lessen with smaller regions of Hsa21 orthologous gene triplication

Improvements in intrinsic mechanical properties, also called material properties, were noted in 16-week-old (4-month-old) Dp1Tyb mice (Thomas et al., 2020). At 16 weeks, there was a sex×genotype interaction for modulus, with control males having a lower modulus than that of all other mice, and both female and male Dp1Tyb mice having a higher modulus than that of their control littermates. Additionally, Dp1Tyb mouse femurs, compared to those from control mice, displayed higher values for yield stress, ultimate stress and resilience. Control, compared to Dp1Tyb, mice had higher values for ultimate strain.

The triplicated segments dividing the Dp1Tyb region all showed a strong sex effect for material properties (Table 7). The Dp9Tyb line displayed sex effects on all intrinsic parameters, with female mice having higher values than those of male mice for all but ultimate strain. The Dp2Tyb line showed interactions for yield stress, ultimate stress and resilience, with male Dp2Tyb and female Dp2Tyb and control mice having significantly higher values than those of male control mice for these parameters, as demonstrated by a post hoc test (Table S1). Additionally, there were differing genotypic effects on ultimate strain and modulus, and sex effects on strain to yield, ultimate strain and modulus (Table 7). Control mice had greater values than those of Dp2Tyb mice for ultimate strain, but Dp2Tyb mice had greater modulus. Female mice had greater values for strain to yield and modulus but lower values for ultimate strain, than those of male mice. In the Dp3Tyb line, female mice had greater values for yield stress, ultimate stress, strain to yield, modulus and resilience than those of male mice, and male mice had greater values for ultimate strain than those of female mice. A genotypic effect was observed for yield stress, where Dp3Tyb mice had greater values than those of control mice.

**Table 7.**
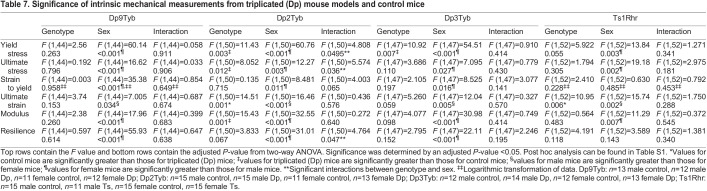
Significance of intrinsic mechanical measurements from triplicated (Dp) mouse models and control mice

To better understand the influence of triplicated genes in Dp3Tyb mice on intrinsic bone parameters, the Dp4Tyb, Dp5Tyb and Dp6Tyb lines were investigated (Table 8). There were only minimal effects in the Dp4Tyb line, with female mice exhibiting higher values for ultimate stress than those of male mice. In the Dp5Tyb line, female mice had greater measurements for yield stress, ultimate stress and modulus than those of male mice. A genotypic effect was observed for ultimate strain, where control mice had greater values than those of Dp5Tyb mice. In the Dp6Tyb line, there was a sex×genotype interaction for modulus, with female Dp6Tyb mice showing greater values than those of female control, male control and Dp6Tyb mice in post hoc tests (Table S1). Additionally, female mice, compared to male mice, had greater values for yield stress, ultimate stress and resilience, while male mice had larger values for ultimate strain than those of female mice.

**Table 8.**
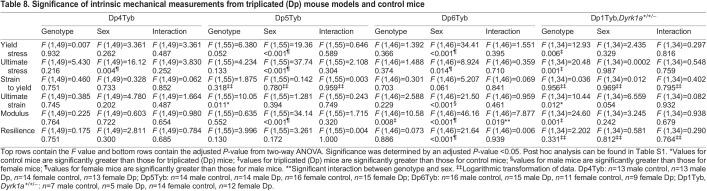
Significance of intrinsic mechanical measurements from triplicated (Dp) mouse models and control mice

A genotypic effect on ultimate strain was observed for the Ts1Rhr line, with Ts1Rhr mice displaying lower values than those of control mice (Table 7). Additionally, the Ts1Rhr line showed sex effects on yield and ultimate stress, ultimate strain and modulus, with female mice having greater measurements than those of male mice for all but ultimate strain. Genotype effects on yield stress, ultimate stress, ultimate strain and modulus appeared to still be present in Dp1Tyb;*Dyrk1a*^+/+/−^ mice, which had higher measurements than those in control mice for all but ultimate strain (Table 8).

## DISCUSSION

### Triplicated Hsa21 orthologous genes interact to cause skeletal phenotypes and, when reduced in number, may have different effects on bone

The manifestation of skeletal defects associated with DS appears to have genetic and sexually dimorphic components. Although three copies of Hsa21 homologous, Mmu16 genes together produce trabecular, cortical and mechanical bone deficits in the Dp1Tyb DS mouse model in a sex-specific manner (Thomas et al., 2020), the contribution of triplicated genes other than *Dyrk1a* has not been elucidated. The analysis of skeletal phenotypes from the mouse mapping panel comprising different sets of triplicated genes demonstrated that, although *Dyrk1a* may influence some bone parameters, other triplicated genes also affect the incidence and severity of DS-related bone phenotypes. Similar to data from the Dp1Tyb line (Thomas et al., 2020), male mice generally had increased trabecular and cortical skeletal measurements compared to those of female mice. Additionally, there appear to be sex effects that interact with triplicated genes that may portend the sexually dimorphic appearance and severity of skeletal deficits in humans with DS.

For trabecular bone, the triplicated genes included in the Dp9Tyb and Dp2Tyb mouse lines improved many trabecular parameters compared to those of euploid littermates, indicating a positive effect of triplicated genes in both regions. Other genotypic effects of three copy Hsa21 orthologous genes on trabecular phenotypes were observed when the Dp5Tyb and Dp6Tyb segments were isolated from the rest of the Hsa21 homologous genes. In post hoc analyses, male, but not female, Dp5Tyb mice had reduced BMD compared to that of control mice.

For cortical bone, Dp9Tyb males and females exhibited generally increased cortical parameters, in addition to their increased trabecular parameters. In Dp2Tyb mice, there were genotype×sex interactions for Tt.Ar, Ma.Ar and Ec.Pm. In contrast to increased trabecular parameters, Dp2Tyb, compared to control mice, showed decreased cortical measurements. Dp3Tyb, Ts1Rhr and Dp5Tyb mice all showed generally decreased cortical parameters compared to those of control mice.

For extrinsic bone properties, some measurements were better in female mice and others were better in male mice. Female mice showed better intrinsic properties than those of male mice, except for ultimate strain, in most of the lines. Most interactive effects on intrinsic measurements were seen in the Dp2Tyb line. The Ts1Rhr line seemed to have extrinsic and intrinsic skeletal phenotypes that bridged differences between the Dp3Tyb and Dp5Tyb lines, suggesting effects from one or more of the 19 additional triplicated protein-coding genes in the Ts1Rhr, but not the Dp5Tyb, regions.

The varying changes in trabecular microarchitecture, cortical geometry and mechanical properties between the mouse mapping panel lines suggest the presence of interactive, additive and compensatory effects with Hsa21 orthologous genes in three copies. However, this study only accounts for the contribution of Hsa21 homologs from Mmu16; the contributions of trisomic homologs from Mmu10 and Mmu17 are unknown and could alter these results. The interaction of triplicated *Dyrk1a* with the other three copy genes found on Mmu16 has been analyzed for hippocampus-dependent memory processes (Duchon et al., 2021). Increased dosage of *Dyrk1a* was found to be important in working memory deficits and increased activity but was modified by other genes on Mmu16 to suppress changes in activity. Genetic interactions of at least two causative and two modifying loci were found to influence recognition memory. Triplicated Mmu16 loci were also analyzed in a panel of triplicated DS mouse models but did not seem to be causative of behavioral deficits (Duchon et al., 2021). In the hippocampal expression analyses, 38-57% of the triplicated genes in a panel of triplicated DS mouse models exhibited dosage compensation and were not significantly differentially expressed.

Similar additive and compensatory dosage effects have been seen in triplicated Hsa21 orthologous gene dosage experiments in zebrafish (Edie et al., 2018). When combinations of mRNA from Hsa21 homologs were injected into zebrafish, some combinations produced additive effects, where the additional mRNA dosage resulted in a significant increase in phenotypic penetrance, while others represented a partial compensatory effect, where the additional mRNA dosage resulted in a significant decrease in phenotypic penetrance. Data from mice and zebrafish, and our current data, suggest that interaction between triplicated Hsa21 orthologous genes and the effect of sex have a complex influence on DS-associated phenotypes.

### Three copies of *Dyrk1a* cause DS-related skeletal differences specific to different bone compartments

Preclinical genetic studies have hypothesized the influence of three copies of *Dyrk1a* on skeletal deficits in DS mouse models. For trabecular measures at 4 months of age, three copies of *Dyrk1a* appear to play a significant role, with a major effect on trabecular bone deficits in male mice. In 16-week-old male Dp1Tyb mice, there were significant reductions in BMD, BV/TV and Tb.Th compared to those in age-matched control mice. Similar reductions were seen in male Dp5Tyb mice that also have three copies of *Dyrk1a* and 11 other genes, although these measurements appeared reduced in magnitude in Dp5Tyb male mice compared to those in Dp1Tyb mice (Table S2). Combining the data from the mouse mapping panel with those from the Dp1Tyb;*Dyrk1a*^+/+/−^ line suggested a direct influence of three copies of *Dyrk1a* and interaction with other genes in the Dp3Tyb region on decreasing Tb.Th in triplicated mice, although triplicated gene(s) in the Dp6Tyb region may result in increased Tb.Th in female Dp6Tyb compared to control mice.

Both male Dp3Tyb and Ts1Rhr mouse models have three copies of *Dyrk1a* but did not show significant differences in trabecular phenotypes at 4 months. It is possible that the 19 protein-coding genes in three copies found on the Ts1Rhr, but not the Dp5Tyb, line modify trabecular phenotypes. Additionally, the normalization of trabecular phenotypes in Dp1Tyb;*Dyrk1a*^+/+/−^ mice indicated that three copies of *Dyrk1a* also influence manifestation of these trabecular deficits in male triplicated mice. However, *Dyrk1a* is likely to interact with a gene(s) in the Dp6Tyb region to fully manifest this deficit in DS mice.

Three copies of *Dyrk1a* may also be involved in some cortical deficits and may interact with other triplicated Hsa21 orthologous genes, but are not sufficient to cause these phenotypes at 4 months of age. Dp1Tyb, Dp3Tyb, Ts1Rhr and Dp5Tyb mice (all with three copies of *Dyrk1a*), compared to control mice, all have significant reductions in Tt.Ar, Ma.Ar, Ps.Pm and Ec.Pm. None of the affected cortical measures, however, were corrected when *Dyrk1a* copy number was normalized in the Dp1Tyb;*Dyrk1a*^+/+/−^ mice. Additionally, the percentage difference in cortical measures between mice with triplicated regions and control littermates within each strain, especially in male mice, is larger in Dp1Tyb compared to Dp3Tyb, Ts1Rhr and Dp5Tyb lines (Table S3). Taken together, these data indicate that *Dyrk1a* or other genes in the Dp5Tyb region may be necessary, but not sufficient, for cortical phenotypes, but, if *Dyrk1a* is causal, other triplicated Hsa21 orthologous genes are involved in DS-associated cortical phenotypes as well. It is likely that interacting and compensatory triplicated Hsa21 orthologous genes affect cortical phenotypes.

As for potential gene interactions and mechanisms, DYRK1A phosphorylates APP and RCAN1, also known as DSCR1 (Ryoo et al., 2008; Jung et al., 2011). *App* is found in three copies in Dp1Tyb and Dp9Tyb mice, and previous studies indicate that changes in *App* dosage negatively impact bone. Male *App*^−/−^ mice displayed decreased BV/TV and Tb.Th, and increased Tb.Sp, at the age of 2 months, which was attributed to increased bone resorption and decreased bone formation (Pan et al., 2018). Tg2567 mice overexpress a mutated human *APP* gene with the Swedish mutation (APPswe), resulting in increased levels of amyloid-beta and amyloid plaques (Hsiao et al., 1996). At the age of 2 months, male Tg2567 mice displayed decreased BV/TV, thought to be due to increased osteoclast activation (Cui et al., 2011). In further study of APPswe in mature osteoblasts, decreased BV/TV, Tb.N and Tb.Sp were observed in TgAPPswe-Ocn mice at the age of 5 months, likely due to increased osteoclast formation and decreased osteoblastogenesis (Xia et al., 2013). *Rcan1* is triplicated in Dp2Tyb mice, and *in vitro* overexpression of *Rcan1* in mouse bone marrow-derived macrophage-like cells resulted in fewer TRAP^+^ multinucleated osteoclasts, indicating that RCAN1 may have a negative effect on osteoclastogenesis (Kim et al., 2016). In primary calvarial osteoblasts, there was significantly increased ALP activity and bone nodule formation, indicating that RCAN1 may have a positive effect on osteoblast function (Kim et al., 2016). Although the effects of *Rcan1* overexpression alone have not been observed in relation to bone phenotypes *in vivo*, we suspect that a similar phenomenon would occur, potentially resulting in increased BMD and other skeletal parameters, and could influence the skeletal differences seen in the Dp2Tyb line.

### Current study compared to previous bone reports in Ts1Rhr and Tg(*DYRK1A*) mice

Previous reports measuring BMD in 16-week-old Ts1Rhr and control mice found no significant differences in BMD, BMC or bone area in areal measures, no differences in regional femur BMD, a small decrease in regional tibia BMD, and a small increase in regional lumbar spine BMD, as quantified by dual-energy X-ray absorptiometry (DXA). These results led the authors to conclude that 31 protein-coding genes in three copies were insufficient to cause a bone density phenotype (Olson and Mohan, 2011). There were also no significant differences between the sexes seen in the previous study, and no further bone quantification of Ts1Rhr mice was done. In the present study, we examined male and female Ts1Rhr mice on a similar genetic background as previously reported (C57BL/6) and also found no differences in femoral BMD in male or female mice between Ts1Rhr and euploid littermate mice at 4 months, although male mice had increased BMD compared to that of female mice. However, when micro-computed tomography (microCT) analyses of geometry and microarchitecture were performed on the cortical and trabecular bone, respectively, we found that Ts1Rhr male and female mice, compared to control mice, at 4 months had significantly reduced cortical bone measurements, similar to those observed in Dp3Tyb mice. We also observed sex effects on trabecular BMD, BV/TV, Tb.N and Tb.Sp. Taken together, these data indicate that measurement of BMD, BMC and bone area via DXA may not accurately show all the parameters that affect bone in DS mouse models or individuals with DS, and more intricate measures of quantification, such as microCT, are necessary to find skeletal differences. By extension, individuals with DS may have skeletal deficits or may be developing bone deficits that are not reflected at early ages or by measurements of BMD. Only through precise examination of bone structure were these developmental differences, and differences between males and females, teased out.

Previous reports on skeletal abnormalities in Tg(*DYRK1A*) mice, compared to control mice, showed differences in BV/TV, Tb.N and Tb.Sp in male and female mice, and Tb.Th in male mice (Lee et al., 2009). The addition of an extra copy (copies) of *DYRK1A* with these results corroborates our finding of the effect of increased *Dyrk1a* copy number on trabecular phenotypes for male mice. Although we did not see trabecular differences in female Dp5Tyb mice, it may be that other genes in the Dp5Tyb region are modulating the effects of three copies of *Dyrk1a*. This could also be true for Tb.N and Tb.Sp in male mice, in which we also do not see any significant differences in Dp5Tyb from control. The only cortical phenotype measured in the aforementioned study was Ct.Th, and no differences were seen. We still observed differences in Ct.Th between Dp1Tyb;*Dyrk1a*^+/+/−^ mice and control mice, and Ct.Th is also the least well correlated phenotype among other cortical phenotypes (Fig. S11). We observed other cortical phenotypes to be affected by an extra copy of *Dyrk1a* or other genes triplicated in the Dp5Tyb region, and similar cortical deficits may have been found in Tg(*DYRK1A*) mice had these analyses been done.

### Hsa21 orthologous genes in three copies may result in improvement in skeletal measurements

Three copies of some genes orthologous to Hsa21 may also improve trabecular and cortical bone phenotypes (Figs 2-4, Tables 1 and 3). Dp9Tyb mice, compared to control mice, with 74 protein-coding genes in three copies, displayed improved trabecular and cortical bone measures. Dosage-imbalanced genes in the Dp9Tyb region that could contribute to these improved phenotypes include *Btg3*, *Usp16*, *Bach1*, *Tiam1* and *Sod1*. *ANA*, also known as *Btg3*, deficiency was shown to enhance ectopic bone formation (Miyai et al., 2009). Deletion of *Usp16* was shown to lead to decreased mature and progenitor hematopoietic stem cell populations (Gu et al., 2016). *Bach1* inhibition suppressed osteoclastogenesis and upregulated ALP activity and osteoblast mineralization (Wada et al., 2020; Hama et al., 2012; Tian et al., 2019). Knockdown of *Tiam1* was shown to enhance ALP activity (Onishi et al., 2013). *Sod1* knockout in mice had effects on bone formation, and resulted in reduced BMD and stiffness, which may be a result of excessive reactive oxidative species (ROS) (Zhang et al., 2021; Morikawa et al., 2013; Smietana et al., 2010). The aforementioned studies reduced expression of these genes instead of triplicating the expression, and results may vary with differences in the genetic dosage imbalance. For example, overexpression of *Sod1* may improve bone phenotypes through increased elimination of ROS.

Dp2Tyb mice, compared to control littermate mice, displayed increased Tb.N and Ct.Th and reduced Tb.Sp, Tt.Ar, Ma.Ar, Ps.Pm and Ec.Pm. These changes may be due to three copies of *Runx1* and/or *Rcan1*. *Runx1* overexpression has rescued bone loss in ovariectomized mice and enhances osteogenic differentiation (Ji et al., 2017; Luo et al., 2019; Tang et al., 2021). As described earlier, increased expression of *Rcan1* could increase osteoblast function while decreasing osteoclastogenesis, leading to increased bone mass. Additionally, increased copy number of four interferon receptor genes – *Ifnar1*, *Ifnar2*, *Ingr2* and *Il10rb* – may disrupt osteoclast function, as interferon signaling components are involved in bone homeostasis (Place et al., 2021). It will be interesting to examine whether the overexpression of these genes improves trabecular deficits and whether associated mechanisms could be used to improve diverse types of bone abnormalities.

Female Dp6Tyb mice appear to have increased Tb.Th compared to that of control mice. In the Dp6Tyb region, *Pcp4* is naturally overexpressed during the differentiation of osteoblasts, and its induced overexpression *in vitro* was shown to enhance Alizarin Red staining intensity, alkaline phosphatase activity, calcium deposition, and expression of osteocalcin and bone sialoprotein, all indications that osteoblast function is increased (Meng et al., 2020; Xiao et al., 2008). Triplicated *Dyrk1a* could interact with *Pcp4* or other triplicated genes in the Dp6Tyb region to exacerbate trabecular deficits.

### Sexual dimorphism in triplicated DS model mice

Almost all trabecular and cortical skeletal parameters were increased in male compared to female mice, except for TMD. When there was a sex×genotype interaction, male mice with a triplicated region almost always had greater measurements for the trabecular and cortical skeletal parameters than those of female mice within the same line. The effects of sex on extrinsic parameters were mixed, and female mice had better intrinsic measurements than male mice, except for ultimate strain.

**Fig. 5.**
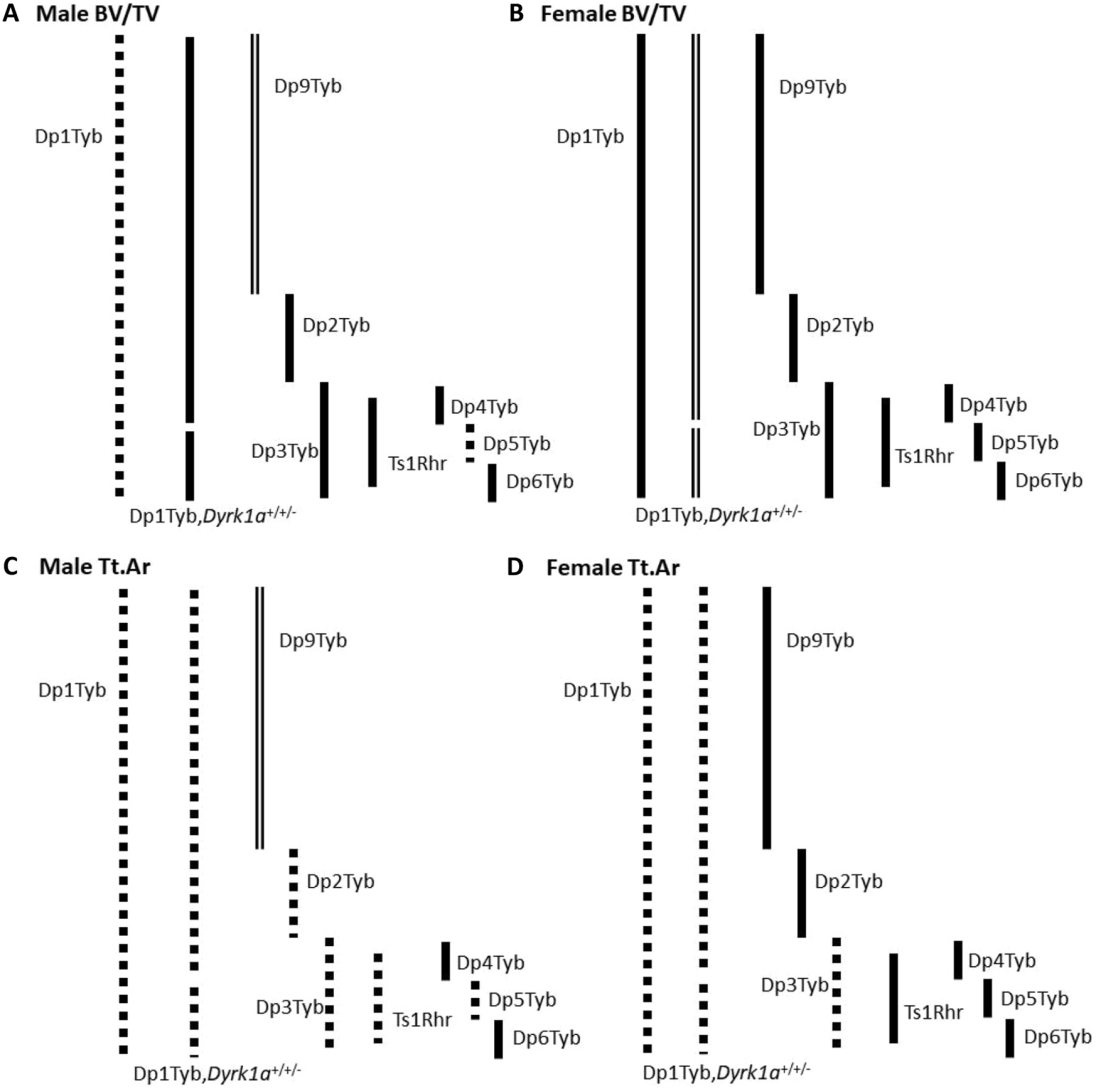
**Representative line images summarizing the significant results on BV/TV and Tt.Ar for all Dp mouse mapping strains.** Solid lines indicate normal compared to control, dotted lines indicate a deficit compared to control, and double lines indicate improvement compared to control. One-way ANOVA; adjusted *P*-value <0.05.

As for the comparisons between male and female mice that include three copies of *Dyrk1a*, there appear to be certain bone phenotypes for which triplicated Hsa21 orthologous genes only affect certain skeletal traits in male or female mice. Overall, it appears that female mice at 4 months of age with any of the Hsa21 orthologs have similar or less-affected skeletal phenotypes than those of euploid littermate mice, whereas male mice with three copies of genes appear to be more affected. Using BV/TV and Tt.Ar as a representation of trabecular and cortical phenotypes, respectively, in males, Dp5Tyb skeletal deficits most resemble those of Dp1Tyb mice, whereas in females, Dp3Tyb skeletal deficits most resemble those of Dp1Tyb mice, at 4 months (Fig. 5). This sexual dimorphism seen in DS mouse models phenocopies that in humans with DS, where males seem to have more differences in skeletal phenotypes than females compared to the general population at an age of peak bone mass (Thomas et al., 2020; Thomas and Roper, 2021). If analyzed at a later time point (post-peak bone mass), skeletal deficits, like decreased BMD, may appear in female mice, as seen in female humans with DS (Carfì et al., 2017).

### Conclusion

It has been assumed that three copies of genes orthologous to Hsa21 on Mmu16 in mice play an important role in the manifestation of trabecular, cortical and mechanical bone property phenotypes. Our previous work, mostly using male mice, implicated triplicated *Dyrk1a* in architectural and mechanical bone phenotypes. By using the mouse mapping panel, we show that three copies of *Dyrk1a* are essential for some skeletal deficits in mice but may interact with other triplicated Hsa21 orthologous genes to cause trabecular, cortical and mechanical deficits in DS mouse models. Understanding how these triplicated Hsa21 orthologous genes interact at the molecular level is an essential next step for understanding and improving skeletal deficits associated with DS.

## MATERIALS AND METHODS

### Animals

The following mouse strains (*Mus musculus*) were used: Dp(16Lipi-Zbtb21)1TybEmcf (Dp1Tyb), Dp(16Mis18a-Runx1)2TybEmcf (Dp2Tyb), Dp(16Mir802-Zbtb21)3TybEmcf (Dp3Tyb), Dp(16Mir802-Dscr3)4TybEmcf (Dp4Tyb), Dp(16Dyrk1a-B3galt5)5Tyb (Dp5Tyb), Dp(16Igsf5-Zbtb21)6TybEmcf (Dp6Tyb), Dp(16Lipi-Hunk)9TybEmcf (Dp9Tyb) and Dp(16Cbr1-Fam3b)1Rhr (Ts1Rhr or Dp1Rhr), all which have been previously reported (Lana-Elola et al., 2016; Olson et al., 2004). To generate Dp1Tyb;*Dyrk1a*^+/+/−^, Dp1Tyb animals were crossed with mice carrying a loss-of-function allele of *Dyrk1a* (*Dyrk1a*^+/−^) (Fotaki et al., 2002). Breeding of the resulting double mutant resulted in an occasional recombination, which brought both alleles onto the same chromosome. This Dp1Tyb;*Dyrk1a*^+/+/−^ double mutant was then bred against wild-type C57BL/6J mice, giving 50% Dp1Tyb;*Dyrk1a*^+/+/−^ double mutant and 50% wild-type offspring. The latter were used as controls for the double mutant mice. All mice were backcrossed to the C57BL/6J background for at least ten generations and were maintained on this background by crossing heterozygous mutants to C56BL/6J wild-type mice. Mice were bred and maintained in specific pathogen-free conditions at the MRC Harwell Institute using Rat and Mouse No.3 breeding chow (Special Diets Services, UK) and given water *ad libitum*. Mice were housed in cages of three to five animals and randomized at weaning into cages of mixed genotype, so that each cage had both mutants and wild types across several litters. Male and female mice were used for all strains at 16-18 weeks (4 months) of age. Genotyping was carried out by Transnetyx (Cordova, TN, USA) using quantitative real-time PCR (qPCR). For each mutant allele, a custom qPCR assay was established using a forward and reverse primer and a reporter probe (Table S5). Protein-coding genes were defined by the GRCm39 mouse genome assembly; for this reason, gene numbers have changed from previous estimates. Right femurs were dissected from male and female mice at 4 months of age, wrapped in gauze and frozen in phosphate-buffered saline (PBS) for shipment, and stored at −20°C or −80°C until needed for microCT analysis or mechanical testing. All regulated procedures were carried out with approval from a Local Ethical Review Panel and under authority of a Project Licence granted by the UK Home Office, and in accordance with EU Directive 2010/63/EU. Sample sizes were based on effect sizes of previous results (Thomas et al., 2020) and sample numbers are listed in Tables 1-4.

### MicroCT analysis

Bone analysis was performed as described in Stringer et al. (2017). Briefly, femurs were scanned using a high-resolution microCT system (SkyScan 1172, Bruker, Kontich, Belgium) that was calibrated using two cylindrical hydroxyapatite phantoms (0.25 and 0.75 g/cm^3^ CaHA) prior to each scanning session. Hydration of the femurs was maintained while scanning by wrapping them in parafilm. Femurs were scanned from the distal condyle to the third trochanter using 60 kV, 12 µm resolution, 885 ms integration time, Al 0.5 mm filter and an angular increment of 0.7°. Post-scan, the bones were wrapped in PBS-soaked gauze and stored at −20°C or −80°C until mechanical testing. Scans were reconstructed and rotated using NRecon and Dataviewer (SkyScan, Bruker). Reconstructed and rotated bones were then analyzed using a CT analyzer (CTan; SkyScan, Bruker) and MATLAB (MathWorks, Natick, MA, USA). Trabecular region of interest (ROI) was defined as beginning at the end of the distal growth plate, extending 10% of the total bone length, and isolated from the cortical bone using a custom MATLAB code that creates an irregular anatomic ROI 10 pixels away from the endocortical perimeter. The CTan's batch analyzer was used to obtain BMD, BV/TV, Tb.Th, Tb.Sp and Tb.N for trabecular ROIs. Cortical ROI was calculated as a region of seven transverse slices at 60% of the total bone length away from the end of the distal growth plate. Geometric properties, including Tt.Ar, Ma.Ar, Ct.Ar, Ct.Th, Ps.Pm, Ec.Pm and TMD, were obtained using a custom MATLAB code (Berman et al., 2015; Stringer et al., 2017). The threshold used for segmentation of the trabecular bone was 55-255 and 70-255 for cortical bone. In addition, one analyzer performed rotation and ROI determination for the entirety of one mouse strain to limit variability between analyzers.

### Mechanical testing

Mechanical properties were determined as described previously (Thomas et al., 2020). Briefly, three-point bending was performed on a mechanical testing machine while the bones were fully hydrated with PBS (TA ElectroForce 3200, TA Instruments, Eden Prairie, MN, USA). Bones were tested with a 7 mm support span in anterior–posterior direction with the posterior surface in compression. The loading point was placed directly at the midshaft location, the bone was preloaded to establish contact, and then testing occurred at 0.025 mm/s to failure. The yield point was determined using the slope of the stress-strain curve, then implementing the 0.2% offset method. The ultimate point was determined as the maximum force recorded. The failure point was determined as when the bone broke. Whole-bone (extrinsic) properties included yield and ultimate force and displacement, yield and total work, and stiffness, based on the force-displacement curve. Material (intrinsic) properties included yield and ultimate stress and strain, modulus, resilience and toughness based on the stress-strain curve. Cortical geometry was used to normalize the stress-strain curve from the force-displacement curve.

### Correlational analysis

Data Analytics Computing measured the correlation between cortical features in each category of data – male wild type, male triplicated, female wild type and female triplicated – in each test group. The graphs in Fig. S11 show the correlation values with a range of −1 to 1 and visually represent these numbers with circles so that the relationship between values is immediately apparent. Larger circles represent greater correlation between the features. Smaller dots represent decreasing linear relationship. The darker blue color in circles and values indicated a greater degree of linear dependence; circles and values of darker red corresponded with decreasing linear correlation. The smallest dots and values closest to 0 with a hue of gray approach feature independence.

In addition, we looked at the bidirectional correlation between male wild-type and male triplicated features, as well as female wild-type and female triplicated features. The correlation in Fig. S11 was plotted with only circles (size representing magnitude and color indicating the increasing, decreasing or independent correlation) to get a clear understanding of directional correlation – positive or negative – or independence of the feature of one genotype to the features in the other genotype.

### Statistical analysis

Normality of the datasets was assessed via Shapiro–Wilk test using an alpha of 0.05. When datasets violated Gaussian distribution, the dataset was transformed to their logarithmic form and normality was assessed again via Shapiro–Wilk test. The transformed datasets are annotated with ‘^‡‡^’ in the tables. A total of three outliers, determined by the ROUT method (Q=1%; GraphPad Prism), were excluded from the data analysis: one male Ts1Rhr was excluded from the trabecular and cortical datasets, a different male Ts1Rhr was excluded from the intrinsic and extrinsic (mechanical) datasets, and one male Dp5Tyb was excluded from the mechanical datasets. Two-way ANOVA was performed for all parameters (trabecular, cortical and mechanical) to examine the potential effects of sex and genotype and their potential interaction separately in each independently generated strain. If a significant sex effect occurred, the average for all one sex (e.g. male), regardless of genotype, was compared to the average for all the other sex (e.g. female). If a significant genotype effect occurred, the average for all one genotype (e.g. control mice), regardless of sex, was compared to the average for all the other genotypes (e.g. triplicated mice). If a significant interaction occurred, Tukey's post hoc analysis was performed for the two-way ANOVA, comparing each of the four groups using a family-wise alpha threshold of 0.05 (displayed in Table S1). The assumption of equal variance was assessed using the Levene's test for equality of sample error variances. In cases in which the groups did not have equal variances, main effects were confirmed by one-way Welch's *F* statistic, and the Games-Howell test for multiple comparisons was used as a confirmation when a significant interaction occurred (Table S6). For percentage difference comparisons, unpaired two-tailed Student's *t*-test was utilized, comparing either male control to male Dp/Ts or female control to female Dp/Ts (Tables S2-S4). Given the number of tests run, *P*-values for each category of parameters (trabecular cortical, extrinsic and intrinsic) were adjusted using the Benjamini–Hochberg method (An et al., 2013). An adjusted *P*-value <0.05 was considered significant, and adjusted *P*-values >1.000 were reported as 1.000.

## Supplementary Material

10.1242/dmm.049927_sup1 Supplementary information
