# Supplementary material for: Genetic dissection of triplicated chromosome 21 orthologs yields varying skeletal traits in Down syndrome model mice
Source: Dis Model Mech. 2023 Apr 26;16(4):dmm049927. doi: 10.1242/dmm.049927 (PMC10163323; doi:10.1242/dmm.049927)
Supplement: Supplementary information [file dmm-16-049927-s1.pdf]

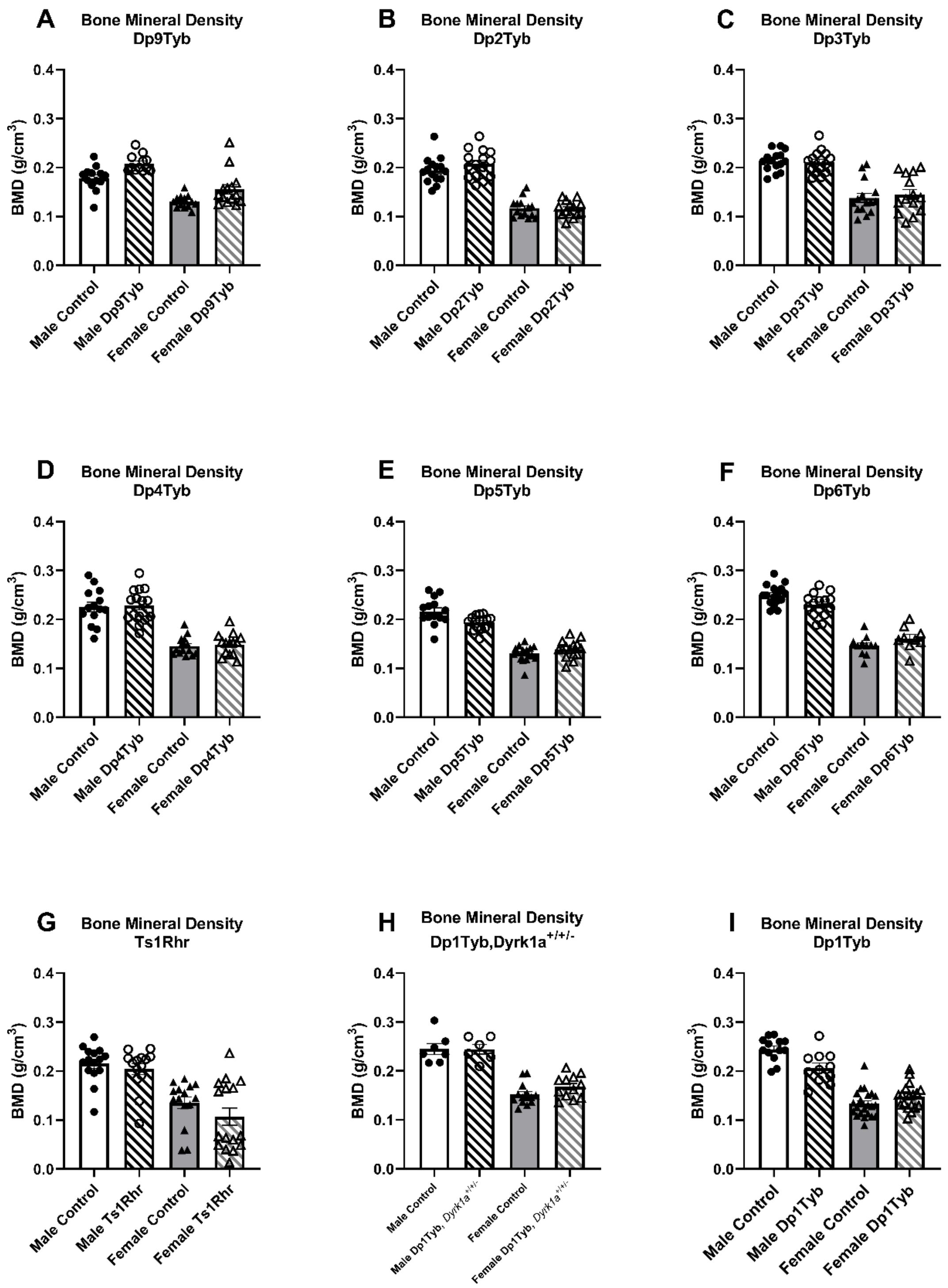

Fig. S1. Bone mineral density measurements in triplicated (Dp) mouse model and control mice. Animal numbers are as listed in Tables 1 and 2. Data are mean ± SEM. Dp1Tyb data comes from Thomas et al. (2020).

Disease Models & Mechanisms • Supplementary information

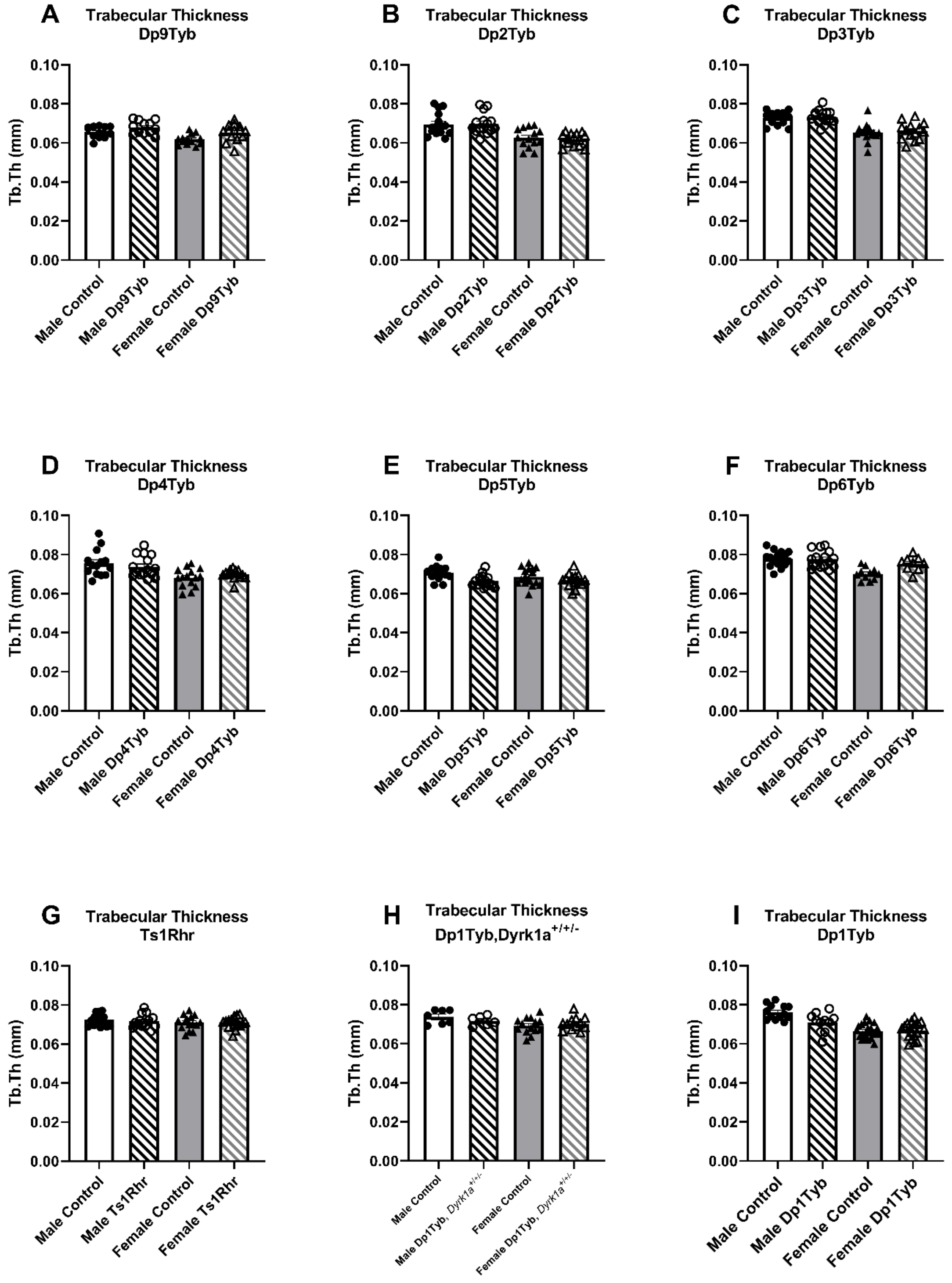

Fig. S2. Trabecular thickness measurements in triplicated (Dp) mouse models and control mice. Animal numbers are as listed in Tables 1 and 2. Data are mean ± SEM. Dp1Tyb data comes from Thomas et al. (2020).

Disease Models & Mechanisms • Supplementary information

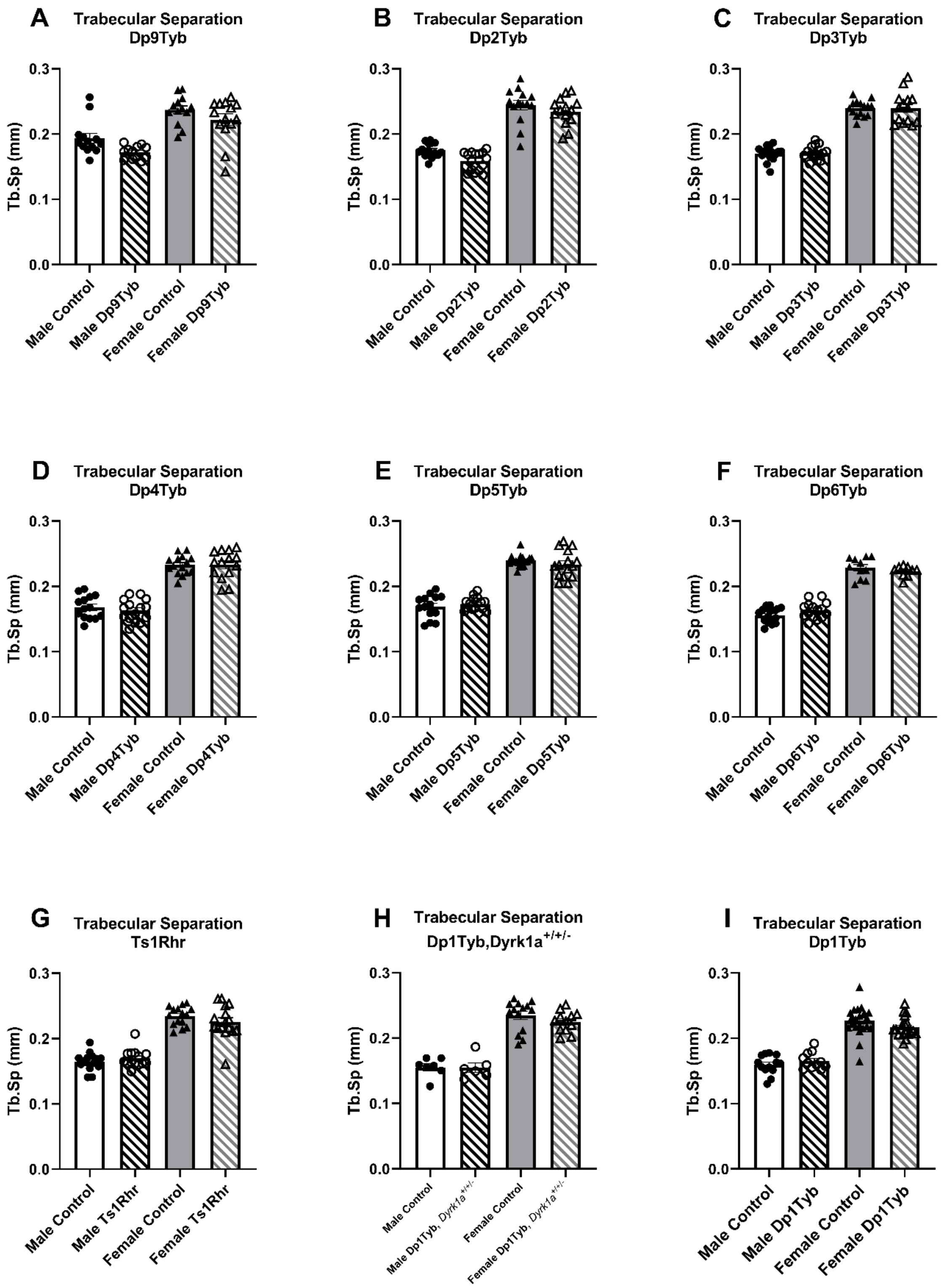

Fig. S3.Trabecular separation measurements in triplicated (Dp) mouse models and control mice. Animal numbers are as listed in Tables 1 and 2. Data are mean ± SEM. Dp1Tyb data comes from Thomas et al. (2020).

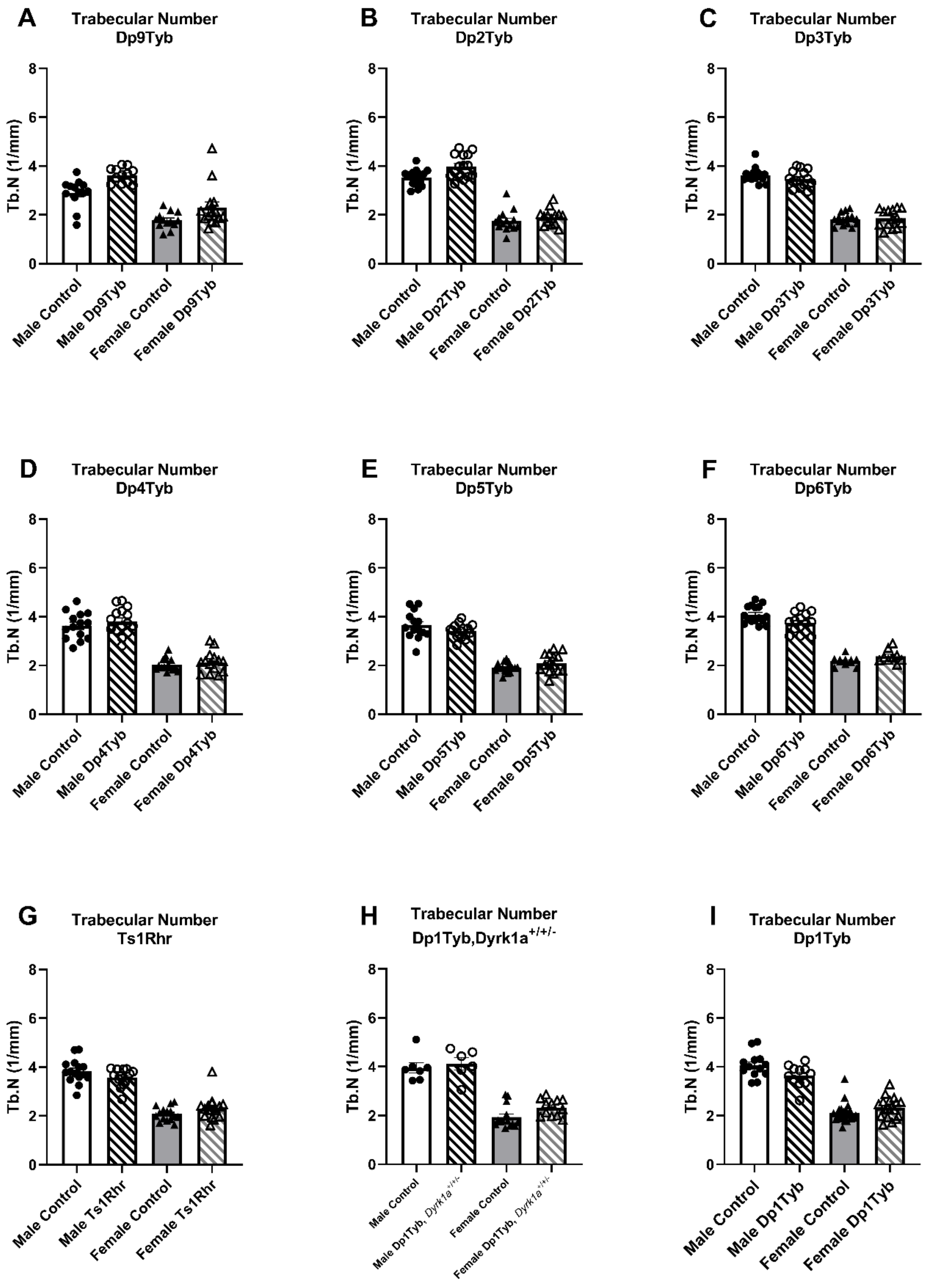

Fig. S4. Trabecular number measurements in triplicated (Dp) mouse models and control mice. Animal numbers are as listed in Tables 1 and 2. Data are mean ± SEM. Dp1Tyb data from Thomas et al. (2020).

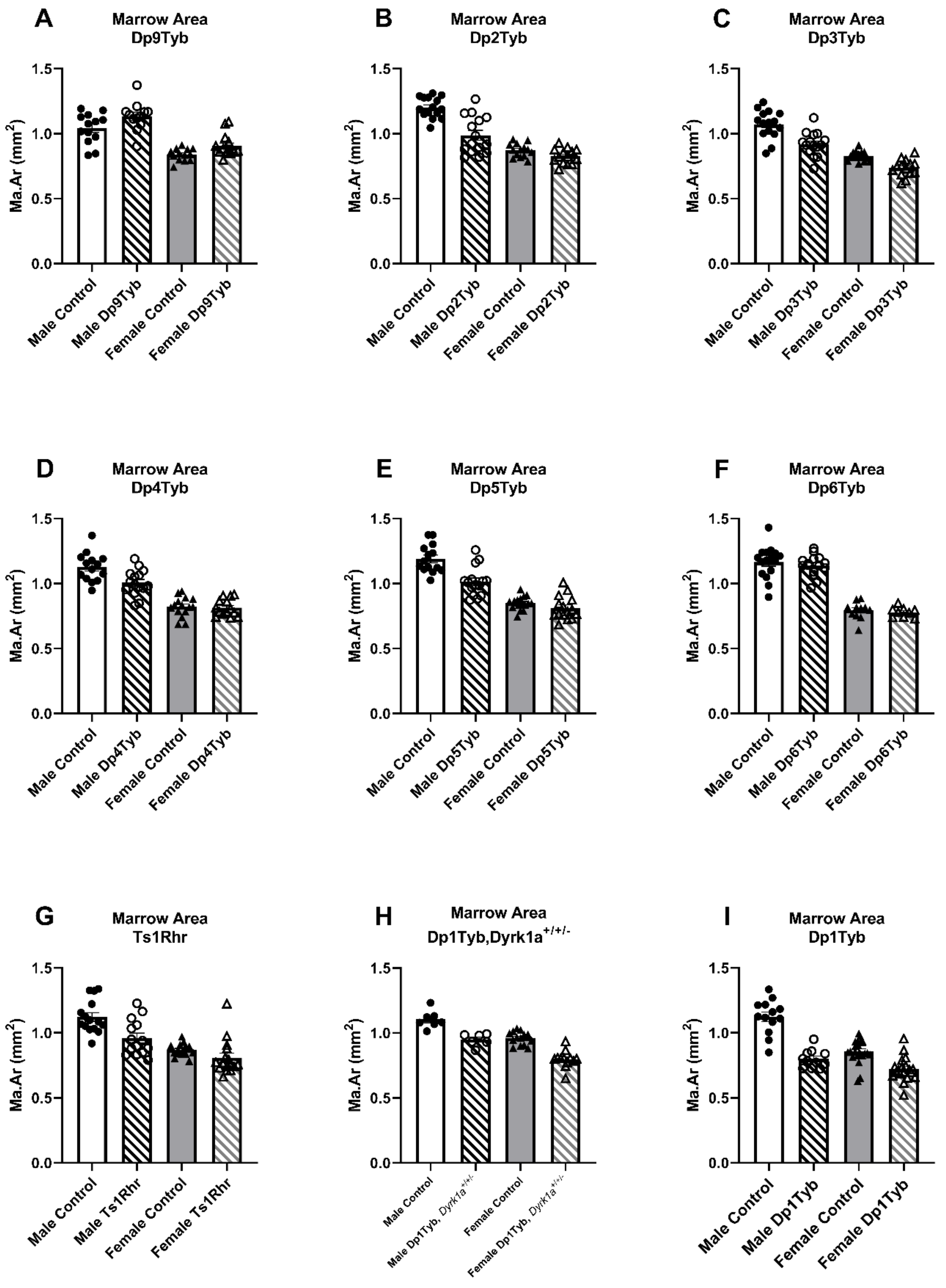

Fig. S5. Marrow area measurements in triplicated (Dp) mouse models and control mice. Animal numbers are as listed in Tables 1 and 2. Data are mean ± SEM. Dp1Tyb data comes from Thomas et al. (2020).

Disease Models & Mechanisms • Supplementary information

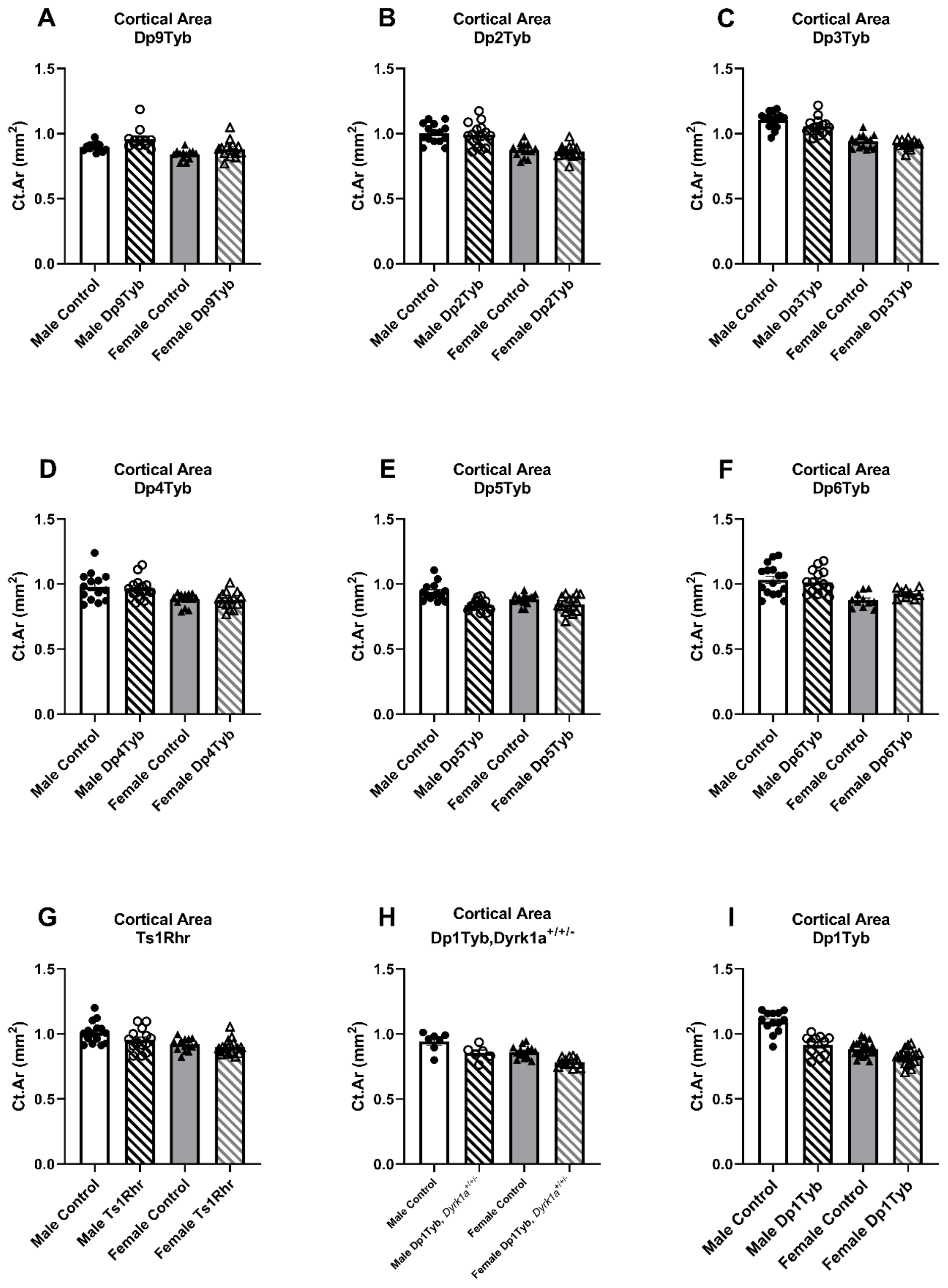

Fig. S6. Cortical area measurements in triplicated (Dp) mouse models and control mice. Animal numbers are as listed in Tables 1 and 2. Data are mean ± SEM. Dp1Tyb data comes from Thomas et al. (2020).

Disease Models & Mechanisms • Supplementary information

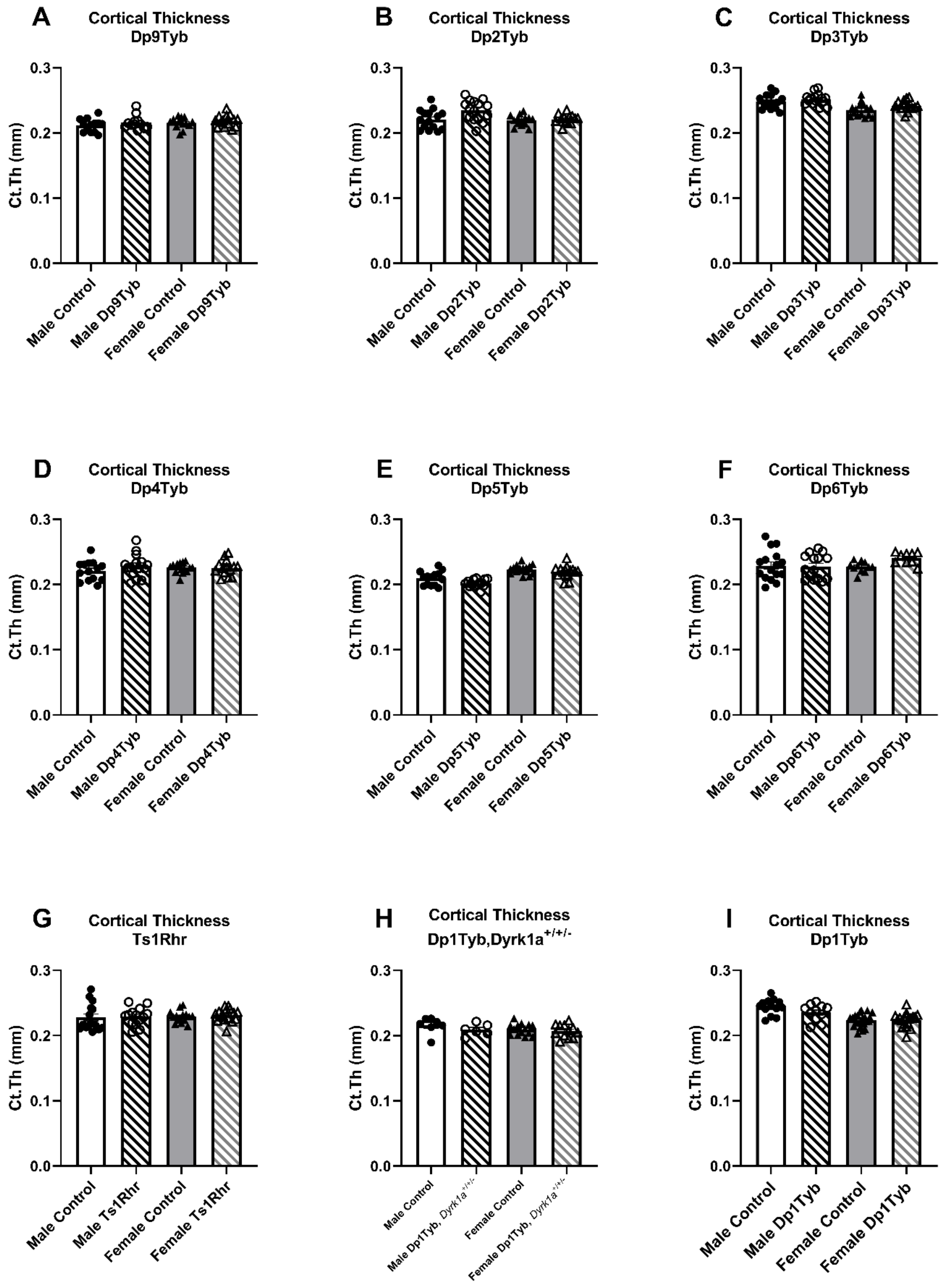

Fig. S7. Cortical thickness measurements in triplicated (Dp) models and control mice. Animal numbers in Tables 2. Data are mean ± SEM. Dp1Tyb data from Thomas et al. (2020).

Disease Models & Mechanisms • Supplementary information

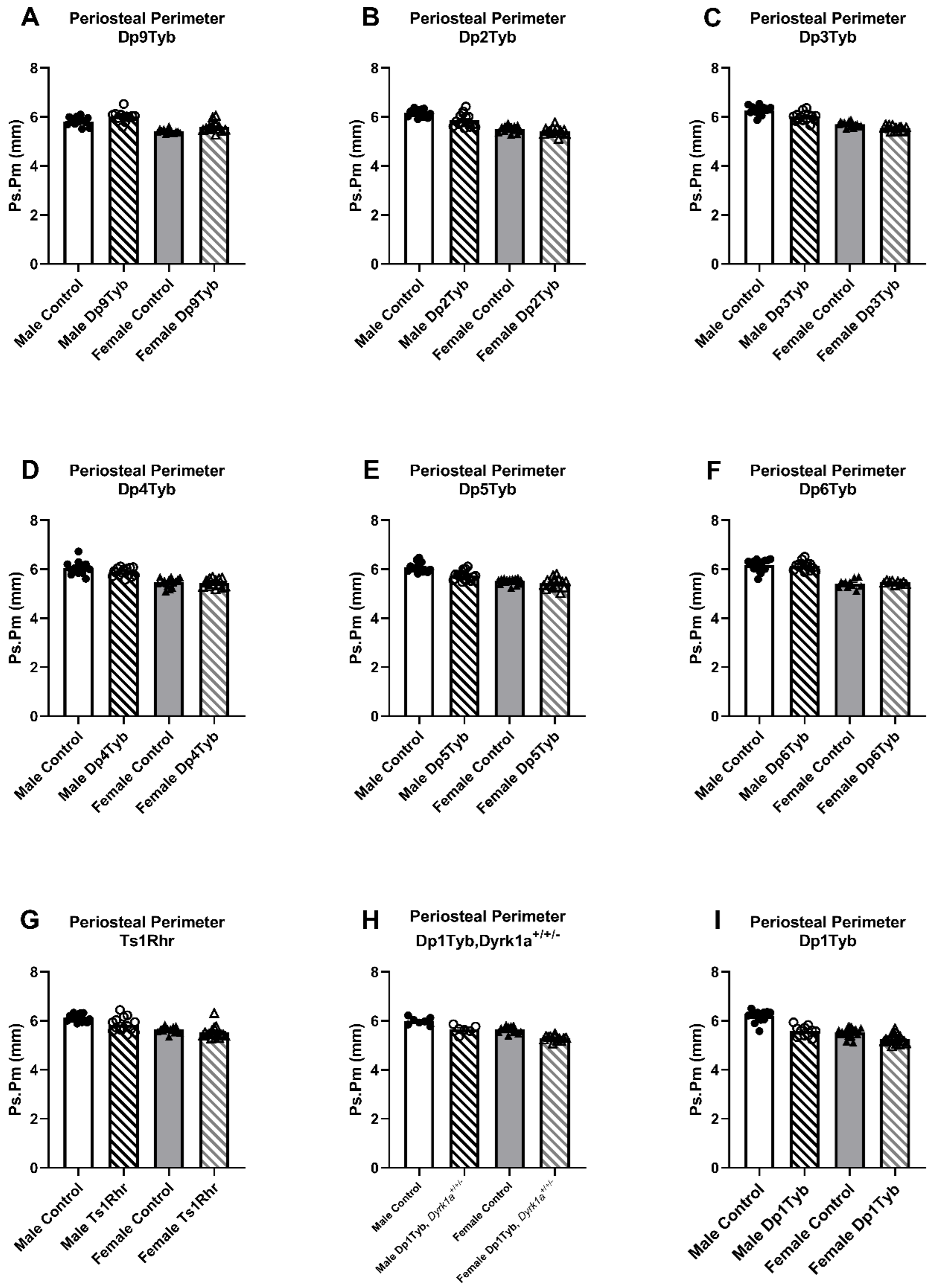

Fig. S8. Periosteal perimeter measurements in triplicated (Dp) mouse models and control mice. Animal numbers are as listed in Tables 1 and 2. Data are mean ± SEM. Dp1Tyb data comes from Thomas et al. (2020).

Disease Models & Mechanisms • Supplementary information

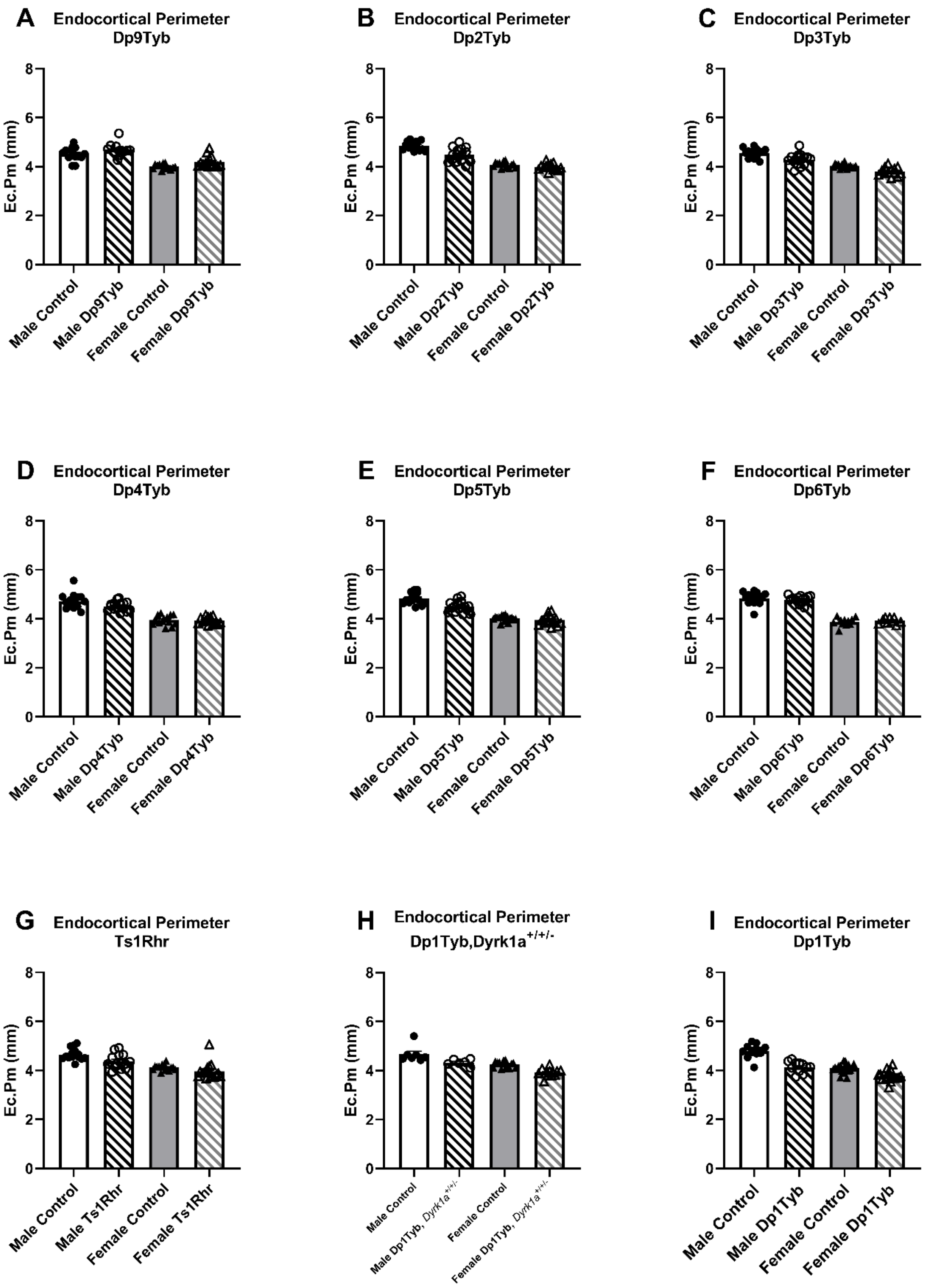

Fig. S9. Endocortical perimeter measurements in triplicated (Dp) mouse models and control mice. Animal numbers are as listed in Tables 1 and 2. Data are mean ± SEM. Dp1Tyb data comes from Thomas et al. (2020).

Disease Models & Mechanisms • Supplementary information

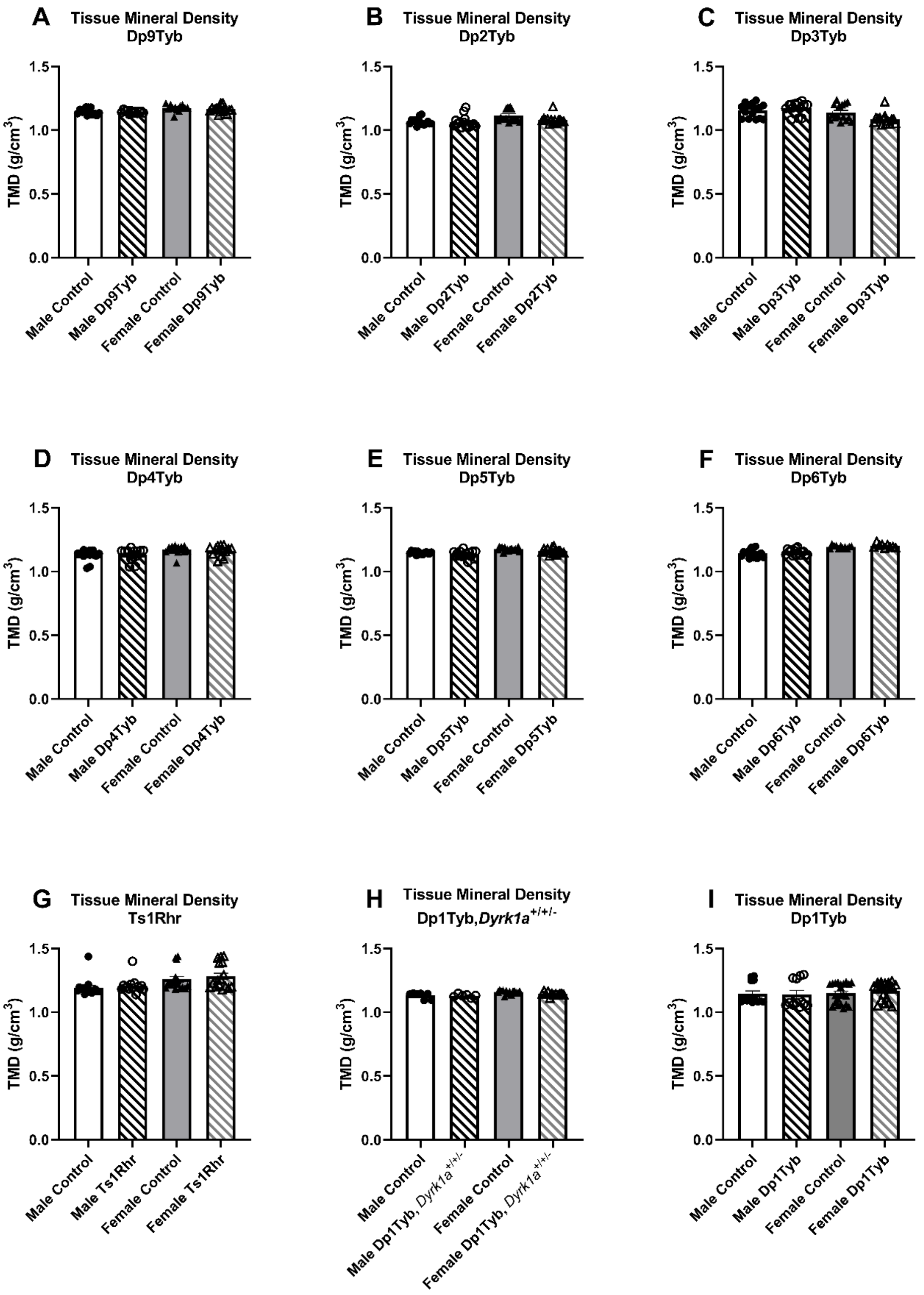

Fig. S10. Tissue mineral density measurements in triplicated (Dp) mouse models and control mice. Animal numbers are as listed in Tables 1 and 2. Data are mean ± SEM. Dp1Tyb data comes from Thomas et al. (2020).

Disease Models & Mechanisms • Supplementary information

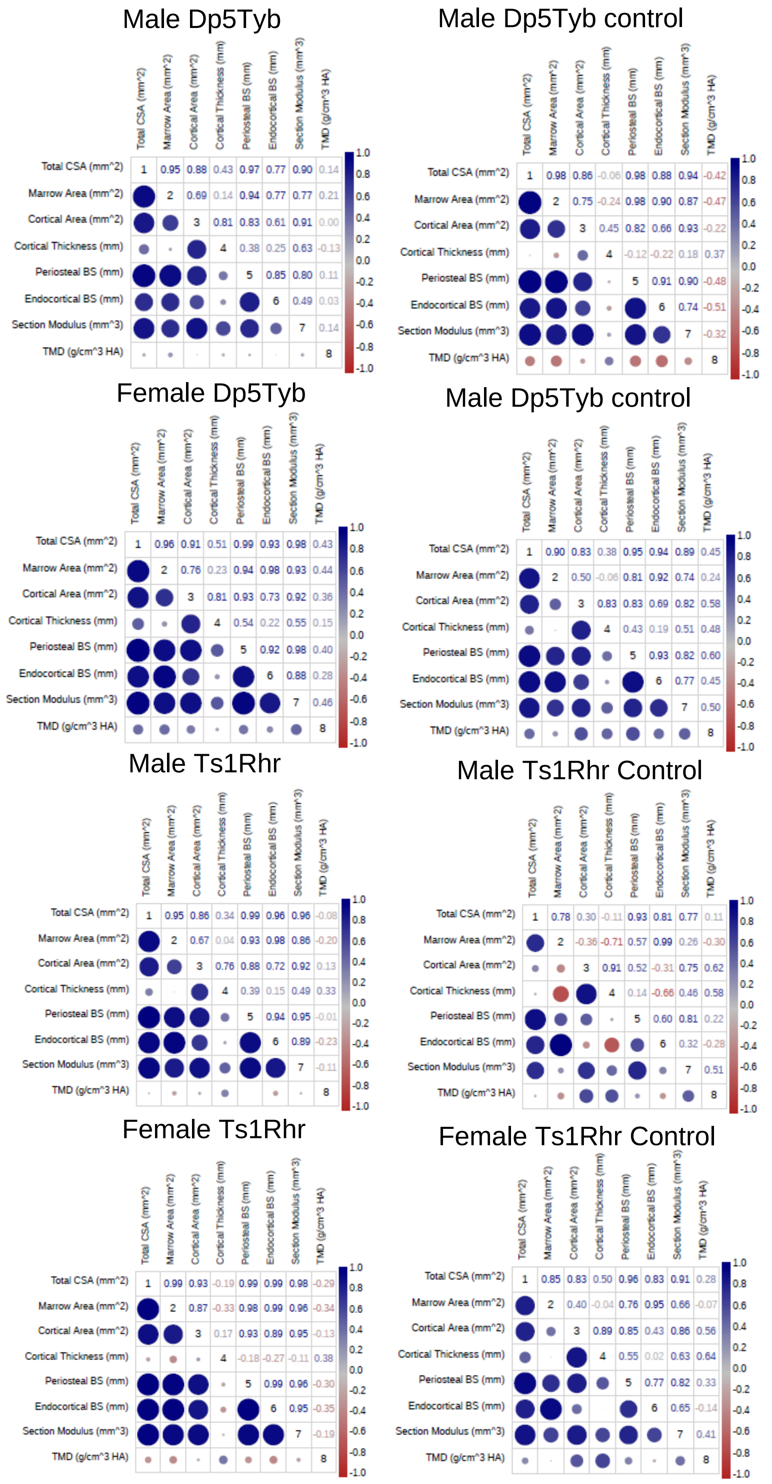

Fig. S11. Correlation between bone parameters of Dp5Tyb and Ts1Rhr male and female mice. Scale of correlation is at the right, with blue as a positive and red as a negative correlation. The larger the circle, the greater the correlation.

Disease Models & Mechanisms • Supplementary information

Table S1. Results of Tukey's post hoc analysis (multiple comparisons) for two-way ANOVAs. This was only performed if there was a significant (adjusted p value < 0.05) interaction on the two-way ANOVA. [a] logarithmic transformation of data. [b] significant Levene's test (unequal variances) and subsequent analysis indicated no significant difference. M = male, F = female, WT = control/ wildtype, Dp = triplicated Dp/Ts strain

	**Dp2Tyb**					
	Tt.Ar	***Ma.Ar***	***Ec.Pm***	***Yield Stress***	***Ultimate Stress***	***Resilience***
M WT vs M Dp	0.0003	<0.0001	<0.0001	0.0006	0.0016	0.0158
M WT vs F WT	<0.0001	<0.0001	<0.0001	<0.0001	0.0010	<0.0001
M WT vs F Dp	<0.0001	<0.0001	<0.0001	<0.0001	0.0002	<0.0001
M Dp vs F WT	0.0002	0.0073	<0.0001	0.0185	0.9673	0.0728
M Dp vs F Dp	<0.0001	0.0001	<0.0001	0.0010	0.8413	0.0804
F WT vs F Dp	0.6889	0.5669	0.6048	0.8560	0.9886	0.9988

	**Dp3Tyb**
	TMD
M WT vs M Dp	0.7818
M WT vs F WT	0.8954
M WT vs F Dp	0.0093
M Dp vs F WT	0.3618
M Dp vs F Dp	0.0005
F WT vs F Dp	0.0565

	**Dp5Tyb**						
	BMD	***BV/TV***	***Tt.Ar***	***Ma.Ar***[a]	***Ct.Ar***	***Ps.Pm***	***Ec.Pm***
M WT vs M Dp	0.0137	0.0283[b]	<0.0001	<0.0001	<0.0001	<0.0001	0.0002
M WT vs F WT	<0.0001	<0.0001	<0.0001	<0.0001	0.0346	<0.0001	<0.0001
M WT vs F Dp	<0.0001	<0.0001	<0.0001	<0.0001	0.0002	<0.0001	<0.0001
M Dp vs F WT	<0.0001	<0.0001	0.0654	<0.0001	0.1077	0.0039	<0.0001
M Dp vs F Dp	<0.0001	<0.0001	0.0011	<0.0001	0.9847	<0.0001	<0.0001
F WT vs F Dp	0.5883	0.8185	0.4037	0.4407	0.2208	0.4387	0.6417

	**Dp6Tyb**				
	BMD	***BV/TV***	***Tb.Th***	***Tb.N***	***Modulus***
M WT vs M Dp	0.0685	0.0684	0.9995	0.029[b]	0.9836
M WT vs F WT	<0.0001	<0.0001	<0.0001	<0.0001	0.0292
M WT vs F Dp	<0.0001	<0.0001	0.4481	<0.0001	<0.0001
M Dp vs F WT	<0.0001	<0.0001	<0.0001	<0.0001	0.0684
M Dp vs F Dp	<0.0001	<0.0001	0.5147	<0.0001	<0.0001
F WT vs F Dp	0.4520	0.2257	0.0215	0.5337	0.002

Disease Models & Mechanisms • Supplementary information

Table S2. Percent differences in trabecular skeletal values of male and female triplicated and control littermate mice. Percent change ([([mean of Dp-mean of Control]/mean of Control)*100]) reported in tables. Red indicates significant deficit; blue indicates significant improvement compared to same-sex control littermates (adjusted $p < 0.05$ via two-tail t test). Adjusted p values reported in Table S4.

	MALE								
	**Dp1Tyb**	**Dp1Tyb, *Dyrk1a*$^{+/+/-}$**	**Dp9Tyb**	**Dp2Tyb**	**Dp3Tyb**	**Ts1Rhr**	**Dp4Tyb**	**Dp5Tyb**	**Dp6Tyb**
BMD	-15.78%	-0.11%	17.03%	6.85%	-0.82%	-5.27%	1.21%	-10.35%	-8.03%
BV/TV	-15.83%	1.30%	29.48%	13.17%	-3.29%	-7.82%	2.71%	-11.63%	-8.72%
Tb.Th	-6.71%	-2.60%	3.64%	0.52%	0.88%	-1.21%	-2.33%	-5.68%	-0.19%
Tb.Sp	3.76%	-0.46%	-11.25%	-9.35%	0.67%	2.94%	-2.87%	2.27%	4.96%
Tb.N	-10.29%	4.16%	25.04%	12.88%	-4.12%	-6.64%	5.14%	-6.36%	-8.35%

	FEMALE								
	**Dp1Tyb**	**Dp1Tyb, *Dyrk1a*$^{+/+/-}$**	**Dp9Tyb**	**Dp2Tyb**	**Dp3Tyb**	**Ts1Rhr**	**Dp4Tyb**	**Dp5Tyb**	**Dp6Tyb**
BMD	10.84%	10.55%	19.26%	-1.31%	4.99%	-21.00%	2.17%	6.59%	10.09%
BV/TV	11.12%	22.32%	37.26%	6.27%	2.84%	11.34%	6.35%	6.60%	17.36%
Tb.Th	0.73%	1.57%	5.61%	-1.22%	1.02%	-0.13%	2.49%	-2.57%	7.41%
Tb.Sp	-4.37%	-4.14%	-6.29%	-4.00%	-0.004%	-3.80%	0.09%	-2.46%	-2.50%
Tb.N	9.97%	20.15%	29.24%	8.78%	2.01%	11.48%	4.33%	9.32%	9.19%

Disease Models & Mechanisms • Supplementary information

Table S3. Percent differences in cortical skeletal values of male and female triplicated and control littermate mice. Percent change ([([mean of Dp-mean of Control]/mean of Control)*100]) reported in tables. Red indicates significant deficit; blue indicates significant improvement compared to same-sex control littermates (adjusted $p < 0.05$ via two-tail t test). Adjusted p values reported in Table S4.

	MALE								
	Dp1Tyb	Dp1Tyb, *Dyrk1a*$^{+/+/-}$	Dp9Tyb	Dp2Tyb	Dp3Tyb	Ts1Rhr	Dp4Tyb	Dp5Tyb	Dp6Tyb
Tt.Ar	-22.64%	-11.99%	7.81%	-10.19%	-9.12%	-10.22%	-6.23%	-12.88%	-1.95%
Ma.Ar	-28.91%	-14.16%	8.80%	-17.78%	-14.11%	-14.39%	-10.57%	-14.72%	-2.26%
Ct.Ar	-16.18%	-9.44%	6.65%	-1.08%	-4.28%	-5.59%	-1.24%	-10.55%	-1.61%
Ct.Th	-3.48%	-3.14%	2.12%	6.32%	1.60%	0.65%	3.39%	-3.72%	-0.59%
Ps.Pm	-9.89%	-5.71%	3.50%	-4.87%	-4.13%	-4.54%	-2.60%	-5.36%	-0.44%
Ec.Pm	-13.55%	-7.34%	4.17%	-7.85%	-5.68%	-5.88%	-4.33%	-6.91%	-1.35%

	FEMALE								
	Dp1Tyb	Dp1Tyb, *Dyrk1a*$^{+/+/-}$	Dp9Tyb	Dp2Tyb	Dp3Tyb	Ts1Rhr	Dp4Tyb	Dp5Tyb	Dp6Tyb
Tt.Ar	-11.54%	-13.12%	6.44%	-3.32%	-6.49%	-4.49%	-0.67%	-4.32%	2.29%
Ma.Ar	-16.09%	-16.78%	7.82%	-5.22%	-11.19%	-6.88%	-0.84%	-4.41%	-1.77%
Ct.Ar	-7.13%	-9.04%	5.07%	-1.42%	-2.34%	-2.23%	-0.52%	-4.23%	5.96%
Ct.Th	-0.01%	-1.31%	1.39%	0.79%	2.51%	1.00%	-0.12%	-1.82%	5.89%
Ps.Pm	-4.94%	-6.26%	3.06%	-1.68%	-2.87%	-2.11%	-0.36%	-1.75%	0.89%
Ec.Pm	-7.77%	-8.13%	4.66%	-2.23%	-5.39%	-3.88%	-0.38%	-2.06%	0.98%

Disease Models & Mechanisms • Supplementary information

Table S4. Results (adjusted p values) of two-tail t tests for trabecular and cortical parameters. Significance was declared with an adjusted p value < 0.05). M = male, F = female, WT = control/wildtype, Dp = triplicated Dp/Ts strain

		Dp1Tyb	Dp1Tyb, *Dyrk1a*$^{+/+/-}$	Dp9Tyb	Dp2Tyb	Dp3Tyb	Ts1Rhr	Dp4Tyb	Dp5Tyb	Dp6Tyb
BMD	M WT vs M Dp	0.014	0.986	0.003	0.249	1.000	0.562	0.836	0.024	0.089
	F WT vs F Dp	0.444	0.135	0.063	0.822	1.000	0.330	0.851	0.394	0.188
BV/TV	M WT vs M Dp	0.015	1.000	0.002	0.073	1.000	0.609	0.837	0.034	0.054
	F WT vs F Dp	0.178	0.077	0.059	0.834	1.000	0.391	0.973	0.371	0.050
Tb.Th	M WT vs M Dp	0.014	1.000	0.068	0.861	1.000	0.478	1.000	0.019	0.920
	F WT vs F Dp	0.689	0.471	0.097	0.791	1.000	0.937	1.000	0.368	0.113
Tb.Sp	M WT vs. M Dp	0.304	1.000	0.018	0.007	0.974	0.578	0.764	0.482	0.100
	F WT vs F Dp	0.189	0.299	0.180	0.748	0.999	0.295	0.976	0.301	0.341
Tb.N	M WT vs M Dp	0.059	1.000	0.004	0.015	0.829	0.316	1.000	0.211	0.045
	F WT vs F Dp	0.272	0.129	0.068	1.000	0.938	0.538	0.854	0.556	0.113
Tt.Ar	M WT vs M Dp	<0.001	0.007	0.068	0.001	0.002	0.009	0.105	<0.001	1.000
	F WT vs F Dp	<0.001	<0.001	0.043	0.226	<0.001	0.191	1.000	0.217	0.746
Ma.Ar	M WT vs M Dp	<0.001	0.004	0.085	<0.001	0.002	0.008	0.030	<0.001	0.986
	F WT vs F Dp	<0.001	<0.001	0.035	0.236	<0.001	0.239	1.000	0.178	0.588
Ct.Ar	M WT vs M Dp	<0.001	0.052	0.046	0.723	0.058	0.101	0.734	<0.001	0.986
	F WT vs F Dp	0.001	<0.001	0.068	0.651	0.178	0.351	0.997	0.305	0.082
Ct.Th	M WT vs M Dp	0.108	0.297	0.288	0.024	0.284	0.822	0.277	0.019	0.860
	F WT vs F Dp	0.997	0.466	0.393	0.556	0.122	0.510	0.943	0.165	0.0158
Ps.Pm	M WT vs M Dp	<0.001	0.007	0.101	0.001	0.001	0.006	0.107	<0.001	0.860
	F WT vs F Dp	<0.001	<0.001	0.080	0.231	<0.001	0.343	1.000	0.242	0.667
Ec.Pm	M WT vs M Dp	<0.001	0.048	0.113	<0.001	0.004	0.016	0.131	<0.001	1.000
	F WT vs F Dp	<0.001	<0.001	0.043	0.211	<0.001	0.567	1.000	0.221	0.698

Table S5. qPCR probes utilized by Transnetyx (Cordova, TN, USA) for genotyping

	Probe 1				Probe 2				Probe 3				Probe 4			
	Name	*Forward Primer*	*Reporter*	*Reverse Primer*	*Name*	*Forward Primer*	*Reporter*	*Reverse Primer*	*Name*	*Forward Primer*	*Reporter*	*Reverse Primer*	*Name*	*Forward Primer*	*Reporter*	*Reverse Primer*
**Dp1Tyb**	3'i17	CGGGCCTCTTCGCTATTACG	CTGCAAACTCTAAAAGATCCGGC	CTCTCTCCCTGAGTGCATTCTC	5'i16	CCCTAAGTCCTTGTCCCTCACA	CAGTGCAGATCCGGCGCG	GCAGTTGTTTAAACTTCTAGAGAATGAGTTC								
**Dp9Tyb**	3'i17	CGGGCCTCTTCGCTATTACG	CTGCAAACTCTAAAAGATCCGGC	CTCTCTCCCTGAGTGCATTCTC	5'h04	CTTCTCTGGACCAAAGGGTTCTTGACA	CTAGTGGATCTCGAGCC	CTATGGCTTCTGAGGCGGAAAGAACCA								
**Dp2Tyb**	3'h04	CGGTGCGGGCCTCTT	ATTACGCCAGGGCGCG	CCCCACCCAATGTCCAAAGAC	5'i02	GCCTTGACTGAGGACGTTGA	CGCGCCGGATCGAT	GCAGTTGTTTAAACTTCTAGAGAATGAGTTC								
**Dp3Tyb**	3'i02	CGTTGGCCGATTCATTAATGCA	CTTAACCACACCCTTACTCG	GACACACCACATCACTGAAACAG	5'i16	CCCTAAGTCCTTGTCCCTCACA	CAGTGCAGATCCGGCGCG	GCAGTTGTTTAAACTTCTAGAGAATGAGTTC								
**Dp4Tyb**	3'i02	CGTTGGCCGATTCATTAATGCA	CTTAACCACACCCTTACTCG	GACACACCACATCACTGAAACAG	5'c09	GCGTTACACACAGAGCATGAAC	CCGGATCACACTCATGTCG	GGCTTCTGAGGCGGAAAGA								
**Dp5Tyb**	3'c09	CGGGCCTCTTCGCTATTACG	CACAGCTTTGATCCGGCGCG	AGCCAGGCGGTGCTG	5'b18	CGAACAACTCAAGGGAGGAAAGATC	CGCGCCAAGCTTTA	AGAGCAGAATAGCAGTTGTTTAACTTCT								
**Dp6Tyb**	3'b18	CGTTGGCCGATTCATTAATGCA	ATTTGAGCTTTGATCCGGCGCG	CCTTCCTTCATAACTGAGTGTCGTA	5'i16	CCCTAAGTCCTTGTCCCTCACA	CAGTGCAGATCCGGCGCG	GCAGTTGTTTAAACTTCTAGAGAATGAGTTC								
**Ts1Rhr**	Ts1RhrTg	CCTGAAGTCCCG	CACACCATATCT	CATCAATGTATC												
		GATGCCA	GCATCA	TTATCATGTCTTTTCCGGGCT												
**Dp1Tyb, *$Dyrk1a^{+/+/-}$***	3'i17	CGGGCCTCTTCGCTATTACG	CTGCAAACTCTAAAAGATCCGGC	CTCTCTCCCTGAGTGCATTCTC	5'i16	CCCTAAGTCCTTGTCCCTCACA	CAGTGCAGATCCGGCGCG	GCAGTTGTTTAAACTTCTAGAGAATGAGTTC	Dyrk1a-1-KO	GGAAGACAATAGCAGGCATGCT	CTATGGGTCTAGAGCTCATG	GTACTTCATTTCAGTGTCGTGTTTGTT	Dyrk1a-1-WT	GCGTTTCTGAATCAAGCCCAGATA	AAGTGCGGCTGCTTGAGCT	TCATTTCAGTGTCGTGTTTGTTCATG

Table S6. Results of significant Levene's test and subsequent Welch's F statistic and Games-Howell test. M = male, F = female, WT = control/wildtype, Dp = triplicated Dp/Ts strain. Highlighted cells indicate significance was lost with the alternative analysis.

Click here to download Table S6

Disease Models & Mechanisms • Supplementary information
